# Role of VTA dopamine neurons and neuroligin 3 in sociability traits related to nonfamiliar conspecific interaction

**DOI:** 10.1038/s41467-018-05382-3

**Published:** 2018-08-09

**Authors:** Sebastiano Bariselli, Hanna Hörnberg, Clément Prévost-Solié, Stefano Musardo, Laetitia Hatstatt-Burklé, Peter Scheiffele, Camilla Bellone

**Affiliations:** 1 Department of Basic Neurosciences, University of Geneva, 1211 Geneva, Switzerland; 2 Biozentrum of the University of Basel, 4056 Basel, Switzerland

## Abstract

Atypical habituation and aberrant exploration of novel stimuli have been related to the severity of autism spectrum disorders (ASDs), but the underlying neuronal circuits are unknown. Here we show that chemogenetic inhibition of dopamine (DA) neurons of the ventral tegmental area (VTA) attenuates exploration toward nonfamiliar conspecifics and interferes with the reinforcing properties of nonfamiliar conspecific interaction in mice. Exploration of nonfamiliar stimuli is associated with the insertion of GluA2-lacking AMPA receptors at excitatory synapses on VTA DA neurons. These synaptic adaptations persist upon repeated exposure to social stimuli and sustain conspecific interaction. Global or VTA DA neuron-specific loss of the ASD-associated synaptic adhesion molecule neuroligin 3 alters the behavioral response toward nonfamiliar conspecifics and the reinforcing properties of conspecific interaction. These behavioral deficits are accompanied by an aberrant expression of AMPA receptors and an occlusion of synaptic plasticity. Altogether, these findings link impaired exploration of nonfamiliar conspecifics to VTA DA neuron dysfunction in mice.

## Abstract (web summary)

Individuals with autism spectrum disorder have alteration in social and novelty behaviors. Here, Bellone and colleagues show that chemogenetic inhibition of mouse dopamine neurons in the ventral tegmental area can blunt exploration towards unfamiliar conspecifics, and that these behavioral deficits are recapitulated in mice lacking neuroligin3 gene product.

## Introduction

From infancy, we encounter an array of diverse stimuli from the environment. Repeated exposure to a stimulus can result in habituation whereas nonfamiliar stimuli usually increases exploratory behavior. Habituation and novelty recognition allow us focusing attention on what is unknown, promote exploratory behavior, facilitate learning, and are predictive of cognitive function later in life^1^. Several neuropsychiatric disorders are characterized by deficits in habituation and novelty exploration. In autism spectrum disorder (ASD), young patients show prolonged attention to depictions of objects, but reduced attention to social stimuli^2^. Moreover, ASD patients are hyporesponsive to novel visual stimuli and exhibit slowed habituation to faces^3,4^. Such alterations are observed in a significant number of individuals with ASD, as they have been reported in clinical studies using diverse stimuli and read-outs^5–7^. However, the circuits and neuronal mechanisms underlying this specific aspect of the ASD phenotype remain largely unknown.

Dopamine (DA) neurons in the ventral tegmental area (VTA) and substantia nigra pars compacta (SNc) may contribute to the habituation to familiar stimuli and to the exploration of nonfamiliar stimuli. DA neurons increase their activity in response to novel environments^8^, to stimuli of positive or negative value^9^, and to natural rewards^10^. Interestingly, these neurons also respond to nonrewarding novel stimuli and their responses habituate when the stimulus becomes familiar^11,12^. This has led to the proposal that novelty by itself may be rewarding. In rodents, nonfamiliar conspecifics or nonfamiliar objects increase Ca^2+^-transients in VTA DA neurons and this activity is necessary to promote social, but not object exploration^13^. Glutamatergic synapses onto DA neurons undergo several forms of synaptic plasticity that may contribute to the modification of social interactions in response to experience. Specific synaptic adaptations have been described during development, after drug exposure, cue-reward learning, reciprocal social interactions, and after repeated burst stimulation of DA neurons^14–18^. Furthermore, glutamatergic transmission is altered in several ASD animal models^19^, and we have recently shown that deficits in the postnatal development of excitatory transmission onto VTA DA neurons lead to sociability deficits^20^. Notably, several studies highlight decreased social reward processing in patients with ASD^21,22^, and these alterations have been hypothesized to precipitate further developmental consequences in social cognition and communication^23^. Whether specific forms of synaptic plasticity in the VTA are induced by exposure to nonfamiliar stimuli (novelty-induced synaptic plasticity), and whether aberrant plasticity associated with exploration of nonfamiliar conspecific in the VTA is related to the maladaptive responses in ASD mouse models is still largely unknown.

In this study, we parse the response to and the preference for nonfamiliar conspecifics as specific aspects of sociability controlled by DA neurons. We demonstrate that intact VTA DA neuron excitability is necessary to express a preference for nonfamiliar conspecifics but not for nonfamiliar objects. Additionally, we adopt a conditioned place preference protocol, based on interaction with familiar or nonfamiliar conspecific, to demonstrate that VTA DA neuron function underlies the reinforcing properties of social interaction. Mice lacking the ASD-associated synaptic adhesion molecule neuroligin 3 (*Nlgn3*) exhibit aberrant exploration of nonfamiliar conspecifics as well as deficit in habituation processing. These phenotypes are recapitulated by VTA DA neuron-specific down-regulation of *Nlgn3*. Finally, we discovered a form of novelty-induced synaptic plasticity at glutamatergic inputs onto VTA DA neurons that sustains conspecific interactions and is impaired in *Nlgn3* KO and *Nlgn3* VTA DA knockdown mice.

## Results

### VTA DA neurons and exploration of nonfamiliar conspecifics

**Fig. 1:**
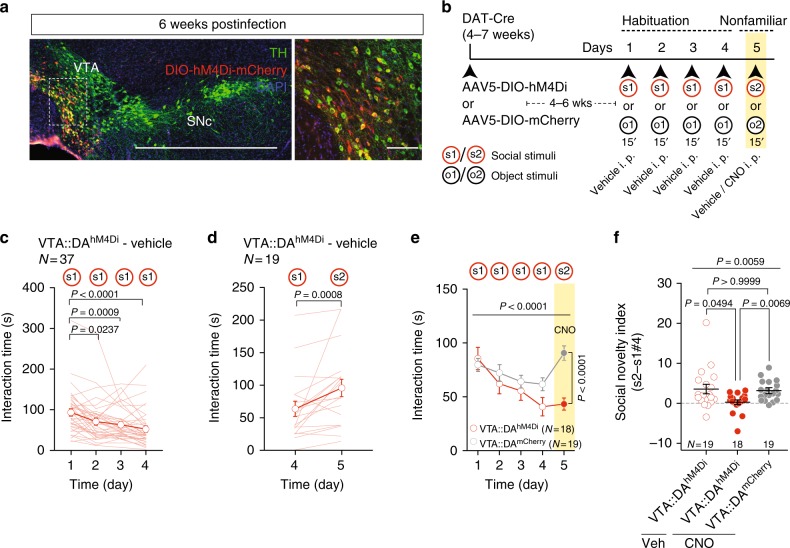
VTA DA neuron excitability controls exploration of nonfamiliar conspecific. **a** Representative images (low and high magnification) of immuno-staining experiments against tyrosine hydroxylase (TH) enzyme (in green) performed on midbrain slices of DAT-Cre mice infected with AAV5-DIO-hM4Di-mCherry (red). Scale bar: 1 mm and 100 μm. **b** Experimental time-course for the habituation/nonfamiliar exploration task. **c** Time course of time interaction for VTA::DA^hM4Di^ mice treated with vehicle during habituation phase. Friedman test (*x*^2^_(4)_ = 32.94, *P* < 0.0001) followed by Dunn’s test for planned multiple comparisons. **d** Graph reporting the time interaction at day 4 with s1 and at day 5 with s2 for VTA::DA^hM4Di^ mice treated with vehicle. Wilcoxon test (*W* = 156). **e** Time interaction over days during the habituation/nonfamiliar exploration task (s1 and s2 are nonfamiliar conspecific stimuli presented at day 1–4 and 5, respectively) for VTA::DA^hM4Di^ and VTA::DA^mCherry^ mice treated with CNO. Repeated measures (RM) two-way ANOVA (time main effect: *F*_(4,140)_ = 12.38, *P* < 0.0001; virus main effect: *F*_(1,35)_ = 3.13, P = 0.0854; time × drug interaction: *F*_(4,140)_ = 9.32, *P* < 0.0001) followed by Bonferroni post hoc test. **f** Social novelty index calculated from VTA::DA^hM4Di^ treated with vehicle, VTA::DA^hM4Di^ and VTA::DA^mCherry^ both treated with CNO. Kruskal–Wallis test (*K*_(3)_ = 10.26, *P* = 0.0059) followed by Dunn’s multiple comparisons test. *N* indicates number of mice. Error bars report s.e.m

Mice have been reported to interact with their conspecifics, to habituate upon repeated contact with the same subject, and to exhibit increased exploration when subsequently brought into contact with a nonfamiliar mouse^24^. To examine whether VTA DA neurons regulate exploration of nonfamiliar conspecifics, we examined the behavior of mice in which the inhibitory DREADD (hM4Di)^25^ or mCherry were virally expressed in DA neurons of the VTA (VTA::DA^hM4Di^: AAV5-hSyn-DIO-hM4Di-mCherry or VTA::DA^mCherry^: AAV5-hSyn-DIO-mCherry injected into DAT-Cre mice, Fig. 1a). Virus infusions led to mCherry expression in 50% of TH^+^ (tyrosine hydroxylase, an enzyme necessary for DA synthesis) VTA neurons and in few (2%) of TH^+^ cells in the neighboring substantia nigra pars compacta (SNc; Supplementary Fig. 1a), confirming preferential targeting of the VTA. Application of the hM4Di ligand clozapine-n-oxide (CNO) decreased the neuronal excitability of VTA::DA^hM4Di^ neurons compared to VTA::DA^mCherry^ ex vivo (Supplementary Fig. 1b) and decreases DA release in striatal regions in vivo^26^.

We then assessed the time spent in social interaction upon repeated exposure to the same mouse (habituation) and the subsequent response to a nonfamiliar conspecific (Fig. 1b). To compare between social and nonsocial stimuli, we also examined the behavioral responses to familiar and nonfamiliar objects (Supplementary Fig. 1c–f). When repeatedly exposed to the same mouse (Fig. 1c) or object stimulus (Supplementary Fig. 1c; s1 and o1, respectively), VTA::DA^hM4Di^ animals injected with vehicle show progressive reduction in the interaction with the stimuli over days. This process has been referred to as habituation^27^. After four habituation days, the animals increased their exploratory behavior toward either a nonfamiliar social (s2; Fig. 1d) or a nonfamiliar object stimulus (o2, Supplementary Fig. 1d) at day 5.

To study the role of VTA DA neurons in this behavioral trait, DA neuron excitability was decreased by intra-peritoneal (i.p.) injection of CNO in VTA::DA^hM4Di^ before the exposure to the nonfamiliar stimulus at day 5. VTA::DA^hM4Di^ animals decreased their exploratory behavior toward the nonfamiliar conspecific whereas control VTA::DA^mCherry^ mice treated with CNO showed unaltered stimulus exploration (s2, Fig. 1e, f). Interestingly, when exposed to a nonfamiliar object (o2), both VTA::DA^hM4Di^ and VTA::DA^mCherry^ animals treated with CNO exhibited an increased exploration (Supplementary Fig. 1e, f). Thus, reducing VTA DA neuron excitability specifically alters the exploration of a nonfamiliar conspecific, but not of a nonfamiliar object, suggesting a differential requirement of DA neuron activity for driving exploration of social and inanimate stimuli.

### VTA DA neurons and preference for nonfamiliar conspecifics

**Fig. 2:**
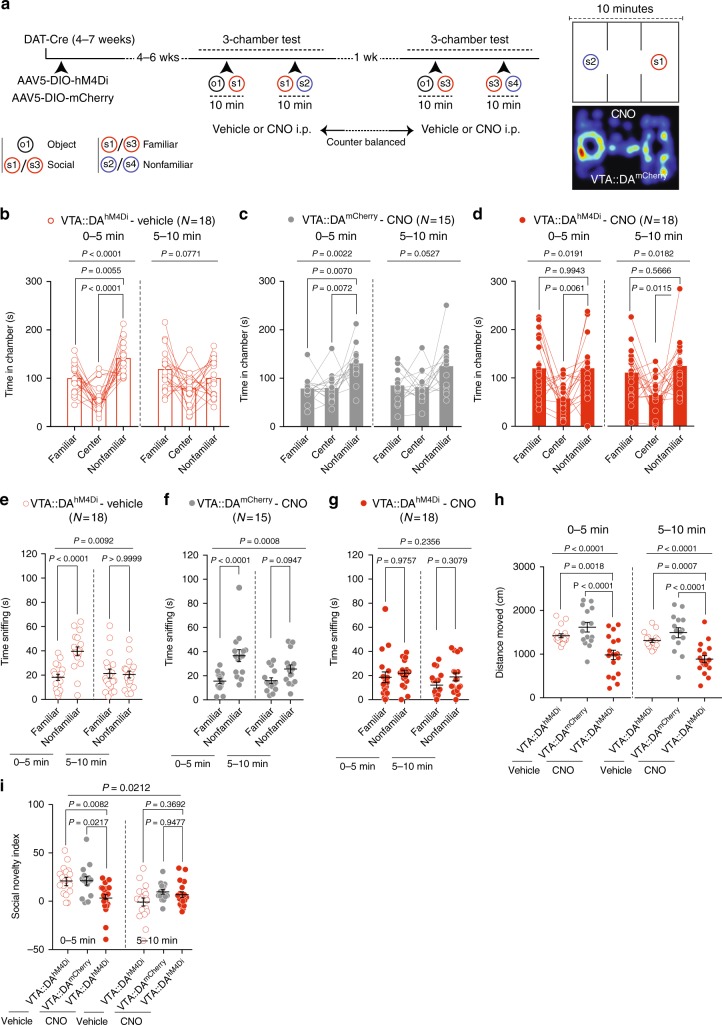
VTA DA neuron excitability controls preference for nonfamiliar conspecific. **a** Left: experimental time-course. Right: apparatus schematic and occupancy plot. **b** Time in chamber for vehicle treated VTA::DA^hM4Di^. RM one-way ANOVA (chamber main effect: *F*_(1.969, 33.48)_ = 21.79, *P* < 0.0001, first 5 mins; chamber main effect: *F*_(1.881, 31.98)_ = 2.825, *P* = 0.0771, last 5 mins) followed by Holm-Sidak post hoc test. **c** Time in chamber for CNO treated VTA::DA^mCherry^. RM one-way ANOVA (chamber main effect: *F*_(1.645, 23.03)_ = 8.959, *P* = 0.0022, first 5 mins; chamber main effect: *F*_(1.545, 21.63)_ = 3.665, *P* = 0.0527, last 5 mins) followed by Holm-Sidak post hoc test. **d** Time in chamber for CNO treated VTA::DA^hM4Di^ mice. RM one-way ANOVA (chamber main effect: *F*_(1.494, 25.4)_ = 5.248, *P* = 0.0191, first 5 mins; chamber main effect: *F*_(1.663, 28.27)_ = 5.006, *P* = 0.0182, last 5 mins) followed by Holm-Sidak post hoc test. **e** Time sniffing for vehicle treated VTA::DA^hM4Di^. RM two-way ANOVA (stimulus main effect: *F*_(1,34)_ = 7.634, *P* = 0.0092; time main effect: *F*_(1,34)_ = 9.617, *P* = 0.0039; time × stimulus interaction: *F*_(1,34)_ = 18.41, *P* = 0.0001) followed by Bonferroni post hoc test. **f** Time sniffing for CNO treated VTA::DA^mCherry^. RM two-way ANOVA (stimulus main effect: *F*_(1,28)_ = 13.96, *P* = 0.0008; time main effect: *F*_(1,28)_ = 5.028, *P* = 0.0330; time × stimulus interaction: *F*_(1,28)_ = 5.629, *P* = 0.0248) followed by Bonferroni post hoc test. **g** Time sniffing for CNO treated VTA::DA^hM4Di^ mice. RM two-way ANOVA (stimulus main effect: *F*_(1,34)_ = 1.458, *P* = 0.2356; Time main effect: *F*_(1,34)_ = 4.806, *P* = 0.0353; time × stimulus interaction: *F*_(1,34)_ = 0.6434, *P* = 0.4281) followed by Bonferroni post hoc test. **h** Distance moved. One-way ANOVA (group main effect: *F*_(2, 48)_ = 12.86, *P* < 0.0001, first 5 mins; group main effect: *F*_(2, 48)_ = 15.01, *P* < 0.0001, last 5 mins) followed by Bonferroni post hoc test for planned comparisons. **i** Social novelty index. RM two-way ANOVA (time main effect: *F*_(2,48)_ = 10.54, *P* = 0.0021; group main effect: *F*_(2,48)_ = 35.503, *P* = 0.0212; time × group interaction: *F*_(2,48)_ = 6.23, *P* = 0.0039) followed by Bonferroni post hoc test for planned comparisons. *N* indicates number of mice. Error bars represent s.e.m

To assess the role of VTA DA neuron excitability in mediating the exploration of a nonfamiliar conspecific over an inanimate object or a familiar conspecific stimulus, VTA::DA^hM4Di^ and VTA::DA^mCherry^ mice were subject to the three-chamber test^28^ under vehicle and CNO conditions. The test was performed twice: first, animals received either vehicle or CNO and, after 1 week of washout, the test was repeated and the pharmacological treatment was counterbalanced (Fig. 2a). To monitor potential off target effects of CNO, we also included VTA::DA^mCherry^ mice treated with CNO as controls. During the task, test mice were given a choice between an object (o1) versus a nonfamiliar mouse (s1 or s3) and subsequently a choice between a familiar (second exposure to s1 or s3) versus a nonfamiliar conspecific (s2 or s4).

Previous studies define sociability in this assay as longer time spent in the chamber with the same-sex nonfamiliar mouse rather than in the chamber with the object, and more time spent sniffing the same-sex mouse rather than sniffing the object^29,30^. According to these criteria, VTA::DA^hM4Di^ mice treated with vehicle (Supplementary Fig. 2a, d), VTA::DA^mCherry^ mice treated with CNO (Supplementary Fig. 2b, e) as well as VTA::DA^hM4Di^ mice treated with CNO (Supplementary Fig. 2c, f) exhibited sociability. We observed a decreased distance moved upon CNO-mediated reduction of DA neuron excitability (Supplementary Fig. 2g). However, despite the reduced locomotion, mice still expressed social preference.

In the second phase of the task, we assessed preference for social novelty that was defined as follows: longer time spent in the chamber with the same-sex nonfamiliar rather than in the chamber with the familiar mouse and more time spent sniffing the same-sex nonfamiliar conspecific rather than sniffing the familiar mouse, particularly during the first 5 min of the test^28^. While preference for social novelty was exhibited by VTA::DA^hM4Di^ mice treated with vehicle (Fig. 2b, e) and by VTA::DA^mCherry^ mice treated with CNO (Fig. 2c, f), it was absent in VTA::DA^hM4Di^ mice treated with CNO (Fig. 2d, g). CNO treated VTA::DA^hM4Di^ mice displayed a reduction in distance moved (Fig. 2h). Additionally, to compare preference for social novelty across groups, we calculated a “social novelty index”, as time spent sniffing the nonfamiliar stimulus minus time spent exploring the familiar target, in the first and last 5 min of the assay. We found that the social novelty index was reduced by CNO injections in VTA::DA^hM4Di^ mice compared to both CNO treated VTA::DA^mCherry^ and vehicle treated VTA::DA^hM4Di^ (Fig. 2i). Altogether, these findings indicate that reducing the excitability of DA neurons decreases the exploration of novel social stimuli, when given a choice between nonfamiliar and familiar conspecifics.

### VTA DA neurons and nonfamiliar conspecific reinforcement

**Fig. 3:**
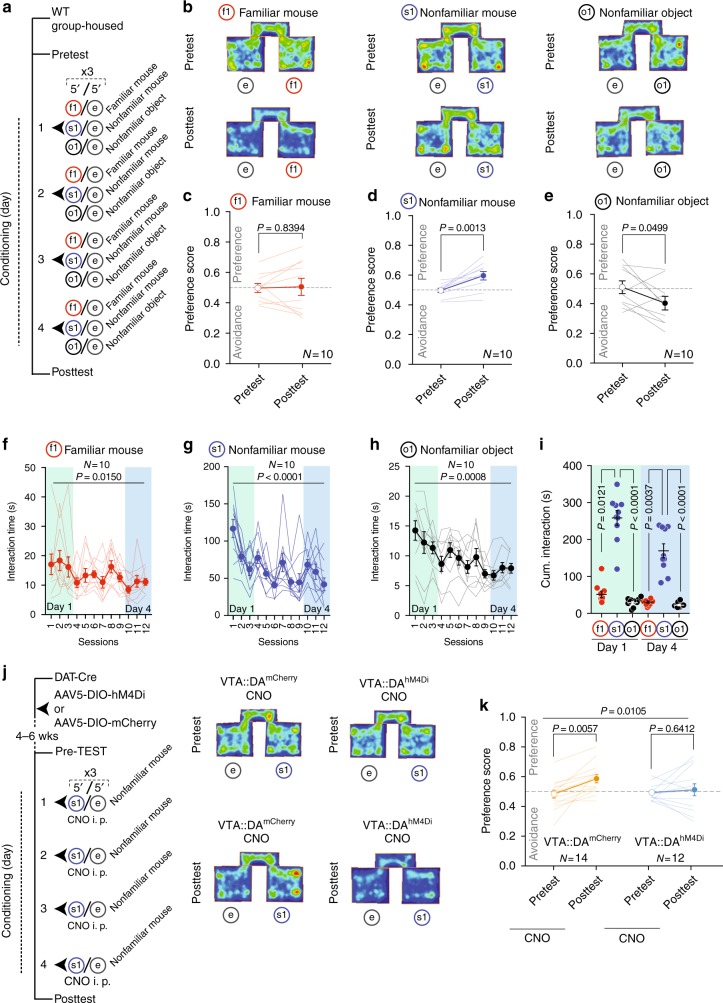
VTA DA neuron excitability mediates the reinforcing properties of nonfamiliar conspecific. **a** Experimental protocol for conditioned place preference with different stimuli. **b** Representative occupancy plots. **c** Scatter plot of preference score measured for familiar mouse pairing during CPP. Paired *t* test (*t*_(9)_ = 0.2086; mean and s.e.m for Pre-TEST: 0.498 ± 0.0298; mean and s.e.m for Post-TEST: 0.506 ± 0.0562). **d** Scatter plot of preference score for nonfamiliar conspecific pairing during CPP. Paired *t* test (*t*_(9)_ = 4.578; mean and s.e.m for Pre-TEST: 0.497 ± 0.0144; mean and s.e.m for Post-TEST: 0.596 ± 0.0285). **e** Scatter plot of preference score for novel object pairing during CPP. Paired *t* test (t_(9)_ = 2.263; mean and s.e.m for Pre-TEST: 0.510 ± 0.0430; mean and s.e.m for Post-TEST: 0.403 ± 0.0455). **f** Time course of interaction during conditioning blocks with a familiar mouse (f1). Friedman test (*P* = 0.0150; *x*^2^_(12)_ = 23.51). **g** Time course of interaction during conditioning blocks with a nonfamiliar conspecific (s1). Friedman test (*P* < 0.0001; *x*^2^_(12)_ = 52.71). **h** Time course of interaction during conditioning blocks with a novel object (o1). Friedman test (*P* = 0.0008; *x*^2^_(12)_ = 31.88). **i** Cumulative interaction during conditioning sessions at day 1 and day 4, respe–Wallis test (K_(6)_ = 46.09, *P* < 0.0001) followed by Dunn’s test for planned comparisons. **j** Left: experimental protocol for VTA::DA^hM4Di^ and VTA::DA^mCherry^ treated with CNO during CPP with nonfamiliar conspecific pairings. Right: representative occupancy plots for CNO treated VTA::DA^mCherry^ and VTA::DA^hM4Di^. **k** Scatter plot of preference score for VTA::DA^mCherry^ treated with CNO during conditioning sessions with a nonfamiliar conspecific (mean and s.e.m for Pre-TEST: 0.4808 ± 0.0267; mean and s.e.m for Post-TEST: 0.5836 ± 0.0275), and scatter plot of preference score for VTA::DA^hM4Di^ treated with CNO during conditioning sessions with a nonfamiliar conspecific (mean and s.e.m for Pre-TEST: 0.4903 ± 0.0162; mean and s.e.m for Post-TEST: 0.5091 ± 0.0428). RM two-way ANOVA (time main effect: *F*_(1, 24)_ = 7.7048, *P* = 0.0105; virus main effect: *F*_(1,24)_ = 0.8678, *P* = 0.3609; time × virus interaction: *F*_(1,24)_ = 3.2861, *P* = 0.0824) followed by Bonferroni post hoc test for planned comparisons. *N* indicates number of mice. Error bars represent s.e.m

To investigate whether nonfamiliar conspecific interactions are reinforcing in mice, we performed a conditioned place preference (CPP) task (modified from 31, 32). Briefly, test mice are housed with familiar mice throughout the protocol. After the Pre-TEST, we performed 4 days of repeated conditioning where wild-type (WT) mice learn to associate one compartment of the apparatus with the presence of either a familiar conspecific (familiar, f1), a nonfamiliar conspecific (s1) or a nonfamiliar object (o1) stimulus, while the other compartment is left empty (Fig. 3a, b). At day 5 (Post-TEST) the preference to explore the two compartments, in absence of any stimulus, was quantified and compared to Pre-TEST. While no significant preference was developed for the familiar conspecifics (Fig. 3c and Supplementary Fig. 3a), mice exhibited preference for the compartment associated with the nonfamiliar conspecifics (Fig. 3d and Supplementary Fig. 3b), and an avoidance for the novel object stimulus associated chamber (Fig. 3e and Supplementary Fig. 3c). Interestingly, across conditioning sessions, we observed habituation to all the stimuli (Fig. 3f–h). However, when the time of interaction with the stimulus during the first and the last day of conditioning was analyzed, we observed a longer interaction with the nonfamiliar conspecifics compared to the other stimuli at either time point (Fig. 3i). These data suggest that a nonfamiliar conspecific remains salient over days and promotes contextual associative learning.

To assess the role of VTA DA neuron excitability in mediating the reinforcing properties of nonfamiliar conspecific interactions, both control VTA::DA^mCherry^ and VTA::DA^hM4Di^ received injections of CNO before each conditioning session and were treated with vehicle before the Post-TEST (Fig. 3j). Control VTA::DA^mCherry^ but not VTA::DA^hM4Di^ mice developed a preference for the compartment associated with the nonfamiliar conspecifics (Fig. 3k and Supplementary Fig. 3d, e). These observations suggest that the excitability of DA neurons mediates both the interaction with nonfamiliar conspecifics as well as the acquisition of nonfamiliar conspecific-induced contextual associations.

### Altered conspecific interactions in *Nlgn3*^*KO*^ mice

**Fig. 4:**
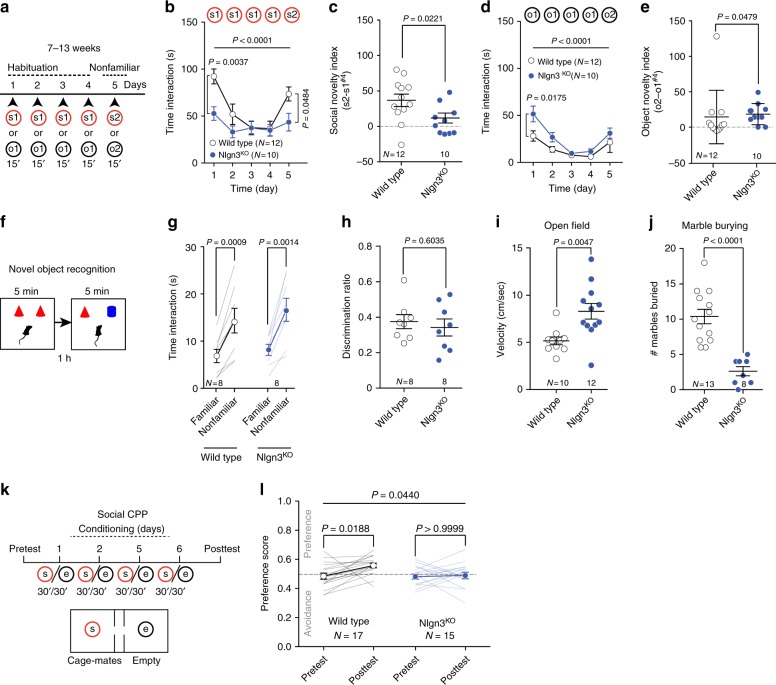
Global knockdown of *Nlgn3* alters sociability and social reward behaviors. **a** Experimental time-course for the habituation/nonfamiliar exploration task. **b** Mean social interaction time for wild type (WT) and *Nlgn3*^*KO*^ mice. RM two-way ANOVA (time main effect: *F*_(4, 80)_ = 20.3, *P* < 0.0001; genotype main effect: *F*_(1, 20)_ = 3.629, *P* = 0.0713; time × genotype interaction: *F*_(4, 80)_ = 6.071, *P* = 0.0003) followed by Bonferroni’s post hoc test. **c** Social novelty index of WT and *Nlgn3*^*KO*^ mice. Unpaired *t* test (t_(20)_ = 2.481). **d** Mean object interaction of WT and *Nlgn3*^*KO*^ mice. RM two-way ANOVA (time main effect: *F*_(4, 80)_ = 17.07, *P* < 0.0001; genotype main effect: *F*_(1, 20)_ = 3.858, *P* = 0.0636; time × genotype interaction: *F*_(4, 80_) = 1.715, *P* = 0.1547) followed by Bonferroni’s post hoc test. **e** Dot plot of object novelty index for WT and Nlgn3^KO^ mice. Mann–Whitney *U* = 30. **f** Schematic of novel object recognition test. **g** Time spent investigating a novel and a familiar object. Paired *t* test (WT: *t*(7) = 5.494. Mean and s.e.m familiar = 6.841 ± 1.41, mean and s.e.m novel = 14.33 ± 2.617. KO: *t*(7) = 5.12. Mean and s.e.m familiar = 8.115 ± 1.186, mean and s.e.m novel = 16.63 ± 2.441). **h** Object discrimination ratio for WT and *Nlgn3*^*KO*^ mice. Unpaired *t* test (*t*_(14)_ = 0.5314). **i** Mean velocity of WT and *Nlgn3*^*KO*^ mice during a 7 min-open field test. Unpaired *t* test (t_(20)_ = 3.178). **j** Number of marbles buried for WT and *Nlgn3*^*KO*^ mice. Unpaired *t* test (*t*_(19)_ = 5.505). (**k**) Schematics of the social conditioned place preference (CPP) test. **l** Scatter plot of preference score measured during the Pre- and Post-TEST for WT (mean and s.e.m for Pre-TEST: 0.4846 ± 0.0209; mean and s.e.m for Post-TEST: 0.5578 ± 0.0158), and *Nlgn3*^*KO*^ mice (mean and s.e.m for Pre-TEST: 0.4809 ± 0.0178; mean and s.e.m for Post-TEST: 0.4886 ± 0.0225). RM two-way ANOVA (time main effect: *F*_(1, 30)_ = 4.422, *P* = 0.0440; genotype main effect: *F*_(1,30)_ = 3.492, *P* = 0.0715; time × genotype interaction: *F*_(1,30)_ = 2.885, *P* = 0.0998) followed by Bonferroni post hoc test for planned comparisons. *N* numbers indicate mice. All error bars are s.e.m

Patients with ASD exhibit slowed habituation to faces^4^ and are less responsive to social reward^22^. Thus, we tested whether a deletion of *Nlgn3* in mice, a category 2 (strong candidate) classified ASD-linked gene (http://gene.sfari.org)^33–35^ encoding a postsynaptic adhesion molecule^36^, might result in deficits in exploration of nonfamiliar conspecifics and in the reinforcing properties of conspecific interaction. Global *Nlgn3*^*KO*^ mice^37^ exhibit reduced ultrasonic vocalization and social memory in male–female interactions as well as altered motor behaviors and olfaction^38–41^. We examined the interaction time upon repeated exposure to a familiar mouse (habituation) and the subsequent response to a nonfamiliar conspecific (Fig. 4a). *Nlgn3*^*KO*^ mice exhibited overall lower interaction times, no significant habituation, and lacked the increased response to nonfamiliar conspecifics seen in Wild Type (WT) littermates (Fig. 4b, c and Supplementary Fig. 4a–d). However, *Nlgn3*^*KO*^ mice showed habituation, increased exploration of nonfamiliar objects (Fig. 4d, e) and preference for nonfamiliar objects in a novel object recognition task (Fig. 4f–h). This indicates that both novelty preference and memory for objects are unaltered. In addition to impaired response to nonfamiliar conspecifics, *Nlgn3*^*KO*^ mutants exhibit alterations in motor activity (Fig. 4i) and marble burying (Fig. 4j). In an olfactory discrimination test^42^, *Nlgn3*^*KO*^ male mice showed normal response and habituation to a social odor (Supplementary Fig. 4e). However, the mutant mice had a significantly decreased response when subsequently presented to a second (novel) social odor (Supplementary Fig. 4e). To further examine conspecific interaction in *Nlgn3*^*KO*^ mice, we tested the reinforcing properties of social interaction^31,32^. When mice are conditioned in a conditioned place preference paradigm with familiar mice, *Nlgn3*^*KO*^ mice did not develop a preference for the social compartments, whereas WT mice did (Fig. 4k, l, and Supplementary Fig. 4f, g). These findings suggest that *Nlgn3*^*KO*^ mice exhibit altered social interactions and defects in social reward behaviors.

### *Nlgn3* loss-of-function in VTA DA neurons alters sociability

**Fig. 5:**
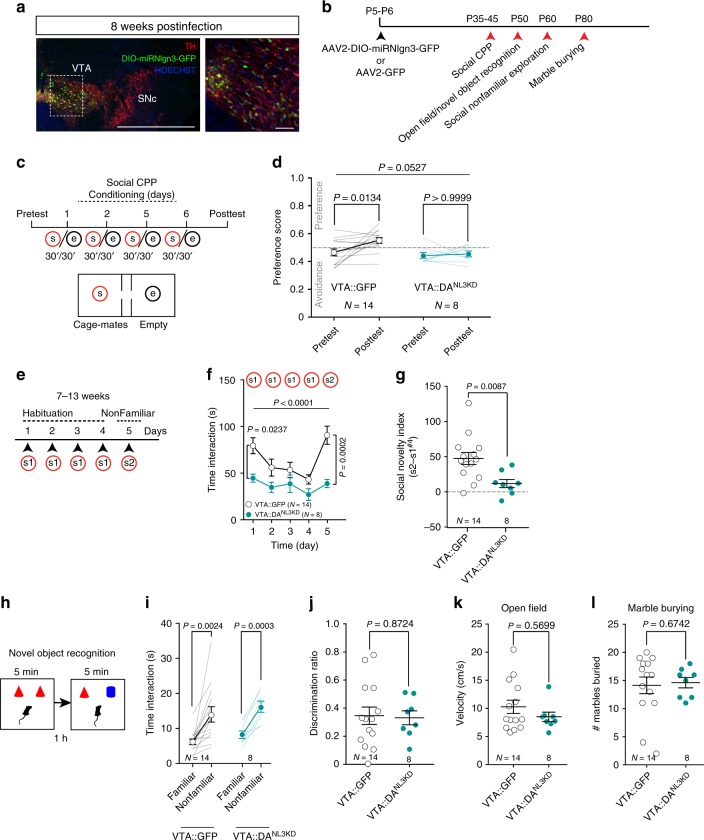
*Nlgn3* in VTA DA neurons is required for social exploration and the reinforcing properties of conspecific interaction. **a** Left: representative image of coronal slice of VTA and SNc from an AAV2 DIO-miR^Nlgn3^-GFP infected DAT-Cre mouse. Right: higher magnification of VTA. Scale bar: 1 mm and 100 μm. **b** Experimental schematic of behavioral test order in VTA-injected mice. **c** Experimental schematic of the social-CPP test. **d** Scatter plot of preference score measured during the Pre- and Post-TEST for VTA::GFP (mean and s.e.m for pre-TEST = 0.4642 ± 0.0247. Mean and s.e.m post-TEST = 0.5526 ± 0.0200), and VTA::DA^NL3KD^ mice (mean and s.e.m for Pre-TEST: 0.4434 ± 0.0218; mean and s.e.m for Post-TEST: 0.4548 ± 0.0214). RM two-way ANOVA (time main effect: *F*_(1, 20)_ = 4.24, *P* = 0.0527; virus main effect: *F*_(1,20)_ = 6.103, *P* = 0.0226; time × virus interaction: *F*_(1,20)_ = 2.527, *P* = 0.1276) followed by Bonferroni post hoc test for planned comparisons. **e** Experimental schematic of the habituation/nonfamiliar exploration task. **f** Mean social interaction plotted for VTA::GFP and VTA::DA^NL3KD^ mice. RM two-way ANOVA (time main effect: *F*_(4, 80)_ = 8.058, *P* < 0.0001, virus main effect: *F*_(1, 20)_ = 9.164, *P* = 0.0067; time × virus interaction: *F*_(4, 80)_ = 3.179, *P* = 0.0178) followed by Bonferroni’s post hoc test. **g** Social novelty index for VTA::GFP and VTA::DA^NL3KD^ mice. Unpaired *t* test (*t*_(20)_ = 2.908). **h** Experimental schematic of novel object recognition test. **i** Time spent investigating a novel and a familiar object. Paired *t* test (VTA::GFP: *t*_(13)_ = 3.763. Mean and s.e.m familiar = 6.199 ± 0.805, mean and s.e.m novel = 14.03 ± 2.188. VTA::DA^NL3KD^: *t*_(7)_ = 6.518. Mean familiar = 8.226, s.e.m ± 1.069, mean novel = 16.2, s.e.m ± 1.582). **j** Discrimination ratio for object discrimination plotted for VTA::GFP and VTA::DA^NL3KD^. Unpaired *t* test (*t*_(20)_ = 0.1627). **k** Mean velocity of VTA::GFP and VTA::DA^NL3KD^ mice during a 7 min open field test. Mann–Whitney *U* = 47. **l** Number of marbles buried plotted for VTA::GFP and VTA::DA^NL3KD^. Mann–Whitney *U* = 49.5. *N* numbers indicate mice. All error bars are s.e.m

The diverse alterations in social but also nonsocial behaviors in *Nlgn3*^*KO*^ mice, indicate that multiple different systems might contribute to their phenotype. To test whether any alterations are due to *Nlgn3* functions in VTA DA neurons we generated microRNA-based knock-down vectors for conditional suppression of *Nlgn3* expression (Supplementary Fig. 5a, b). Cre-dependent AAV-based vectors were injected into the developing VTA of DAT-Cre mice at postnatal days 5–6 and mice were analyzed using a battery of behavioral tests (AAV2-DIO-*miR*^*Nlgn3*^ in DAT-Cre mice: VTA::DA^NL3KD^, Fig. 5a, b, and see Supplementary Fig. 5c for off-target areas affected and Supplementary Fig. 5d, e for further controls). Notably, VTA::DA^NL3KD^ mice exhibited a similar impairment in reinforcing properties of conspecific interaction as the global *Nlgn3*^*KO*^ mice in the conditioned place preference paradigm (Fig. 5c, d, and Supplementary Fig. 5f, g) indicating that *Nlgn3* downregulation in VTA DA neurons is sufficient to mimic this aspect of the global *Nlgn3*^*KO*^ phenotype. Furthermore, when repeatedly exposed to the same and subsequently to a nonfamiliar conspecific, VTA::DA^NL3KD^ mice showed an overall reduction in social exploration and a blunted response to novel conspecific stimuli (Fig. 5e–g, and Supplementary Fig. 5h–k). At the same time, VTA::DA^NL3KD^ mice showed preference for novel objects in the novel object recognition task (Fig. 5h–j). Thus, there is a specific requirement for *Nlgn3* in VTA DA neurons for appropriate exploration of nonfamiliar conspecifics and for the reinforcing properties of social interaction. By contrast, motor activity, marble burying, and social olfaction that are altered in global *Nlgn3*^*KO*^ mice were not modified in the VTA::DA^NL3KD^ mutants (Fig. 5k, l, Supplementary Fig. 5l). Interestingly, we observed that knock-down of *Nlgn3* in VTA-DA neurons of adult mice produced a similar but less pronounced social interaction phenotype as in developing animals, with reduced habituation and reduced response to nonfamiliar conspecifics (Supplementary Fig. 6). Thus, *Nlgn3* expression, in both developing and mature VTA DA circuits, is required for habituation and nonfamiliar conspecific exploration.

### A synaptic signature of saliency detection in VTA DA neurons

**Fig. 6:**
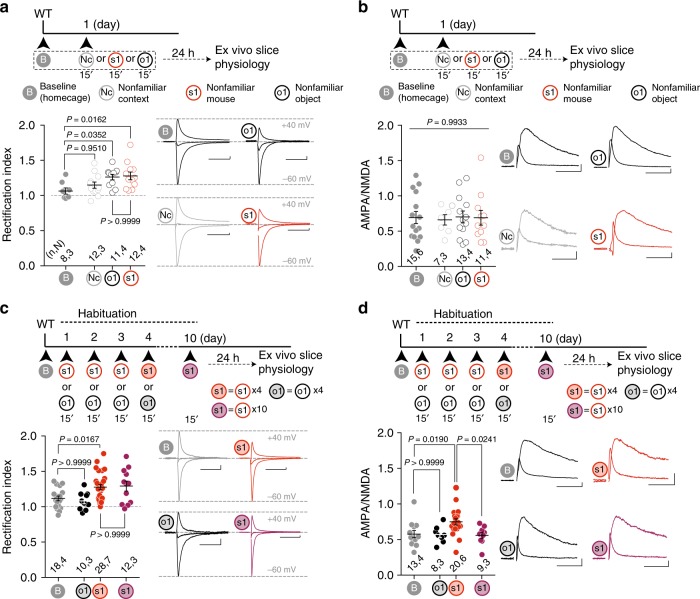
Novelty-induced synaptic plasticity. **a** Top: experimental paradigm. Bottom: scatter plot of rectification index and AMPAR-EPSCs example traces (−60, 0, and 40 mV) recorded from VTA DA neurons at baseline (B, homecage), or 24 h after 15 min of novel context (Nc), nonfamiliar conspecific (s1) or novel object (o1) exposure. One-way ANOVA (*F*_(3, 39)_ = 4.153, *P* = 0.0120) followed by Bonferroni post hoc test for planned comparisons. **b** Top: experimental paradigm. Bottom: scatter plot and example traces of AMPA/NMDA ratio recorded from VTA DA neurons at baseline (B, homecage), or 24 h after 15 min of novel context (Nc), nonfamiliar conspecific (s1) or novel object (o1) exposure. One-way ANOVA (*F*_(3, 42)_ = 0.0287, *P* = 0.9933). **c** Top: experimental paradigm. Bottom: scatter plot and example traces of rectification index recorded from VTA DA neurons at baseline (B), 24 h after four repeated exposures to either a novel mouse (s1) or a novel object (o1) and ten repeated exposures to a nonfamiliar conspecific (s1, bold purple). One-way ANOVA (*F*_(3, 64)_ = 5.149, *P* = 0.0030) followed by Bonferroni post hoc test for planned multiple comparisons. **d** Top: experimental paradigm. Bottom: scatter plot and example traces of AMPA/NMDA ratio recorded from VTA DA neurons at baseline (**b**), 24 h after four repeated exposures to either a nonfamiliar conspecific (s1) or a novel object (o1) and ten repeated exposures to a nonfamiliar conspecific (s1, bold purple). One-way ANOVA (*F*_(3,46)_ = 4.4939, *P* = 0.0076) followed by Bonferroni post hoc test for planned multiple comparisons. *n*,*N* indicates number of cells and mice respectively. Scale bars: 20 msec, 20 pA. Error bars report s.e.m

Several experiences strengthen synaptic transmission at excitatory inputs onto DA neurons and drive the insertion of GluA2-lacking AMPARs, which can be assessed by calculating a rectification index (RI)^17^. We tested whether nonfamiliar exploration induced specific forms of long-lasting synaptic plasticity at excitatory inputs onto DA neurons in the VTA (novelty-induced synaptic plasticity). In WT mice, the RI increased at synapses 24 h after the exploration of either a nonfamiliar mouse or a nonfamiliar object when compared to RI calculated from home caged mice (Fig. 6a). By contrast, the RI was unchanged after the exposure to a new context and AMPA/NMDA ratios were unchanged for any of the above conditions (Fig. 6b). When AMPAR EPSCs were recorded after repeated exposure (over 4 days) to object stimuli, the RI was normalized to control condition (Fig. 6c). A subsequent exposure to a new object (o2) increased the RI (Supplementary Fig. 7a). By contrast, GluA2-lacking AMPARs were detected in mice repeatedly exposed to a nonfamiliar conspecific stimulus (s1) over a 4-day period and were still present at these synapses after 10 days of repeated exposure (Fig. 6c). Remarkably, the AMPA/NMDA ratio was significantly elevated after 4 days of social (s1) repeated exposure relative to baseline but was normalized after 10 days of repeated exposure (Fig. 6d), while the paired-pulse ratio (PPR) remained unchanged throughout (Supplementary Fig. 7b). Taken together, these data indicate that repeated exposure to a nonfamiliar conspecific stimulus, but not an object stimulus, transiently increases synaptic strength (AMPA/NMDA ratio) and produces a stable insertion of GluA2-lacking AMPARs at VTA DA neuron excitatory inputs.

**Fig. 7:**
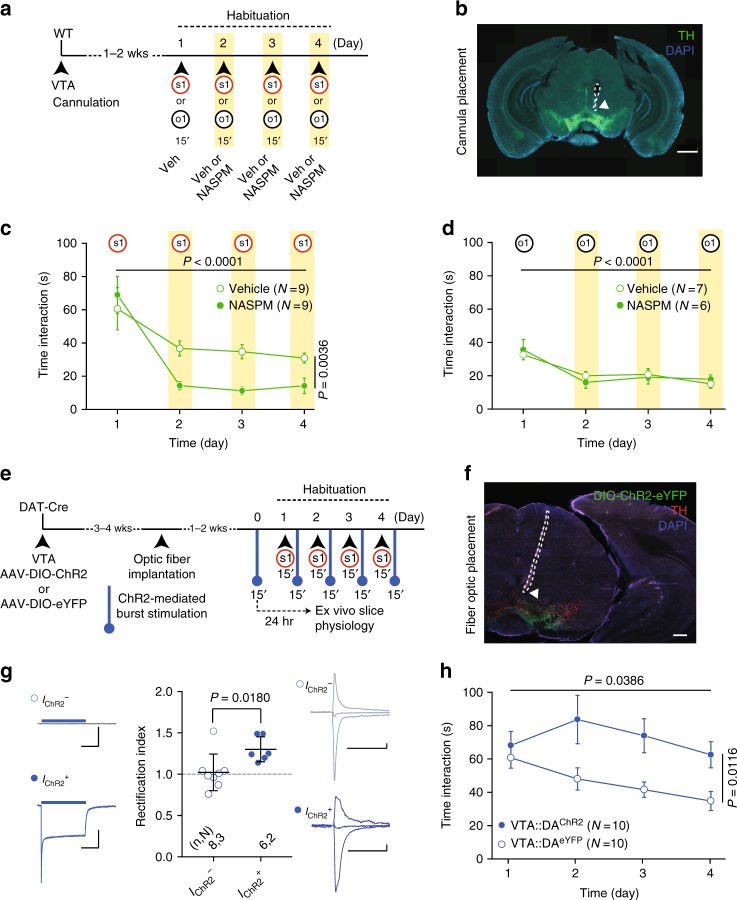
GluA2-lacking AMPAR function controls habituation to nonfamiliar conspecific. **a** Schema of the experimental paradigm. **b** Representative image of cannula placement for NASPM or vehicle infusion (green: TH; blue: DAPI; white arrow indicates cannula tip). **c** Time course of time interaction with a nonfamiliar conspecific (s1) for vehicle or NASPM infused mice at day 2, day 3, and day 4. RM two-way ANOVA (time main effect: *F*_(3, 24)_ = 17.57, *P* < 0.0001; drug main effect: *F*_(1, 8)_ = 16.48, *P* = 0.0036; time × drug interaction: *F*_(3, 24)_ = 3.141, *P* = 0.0439). **d** Time course of time interaction with a novel object (o1) over 4 days for Vehicle and NASPM groups. RM two-way ANOVA (time main effect: *F*_(3, 33)_ = 24.71, *P* < 0.0001; drug main effect: *F*_(1,11)_ = 0.00005, *P* = 0.9942; time × drug interaction: *F*_(3, 33)_ = 1.109, *P* = 0.3595). **e** Experimental paradigm for noncontingent optogenetic stimulation. **f** Representative image of optic fiber placement for DIO-ChR2 expressing mice (red: TH, green: AAV-DIO-ChR2-eYFP, blue: DAPI; white arrow indicates fiber optic tip). **g** Left: example traces of a photocurrent negative (I_ChR2_−) and a photocurrent positive (I_ChR2_^+^) VTA DA neuron. Scale bars: 20 msec, 1 nA. Middle: scatter plot of RI recorded from photocurrent negative (I_ChR_−) and photocurrent positive (I_ChR2_^+^) VTA DA neurons and AMPAR-EPSCs example traces (−60, 0, and 40 mV) recorded from VTA DA neurons. Mann−Whitney test (*U* = 6). Scale bars: 20 msec, 20 pA. **h** Time course over 4 days of time interaction with a nonfamiliar conspecific (s1) for VTA::DA^ChR2^ and VTA::DA^eYFP^ mice with noncontingent optical stimulation. RM two-way ANOVA (time main effect: *F*_(3, 18)_ = 2.9966, *P* = 0.0386; virus main effect: *F*_(1, 18)_ = 7.9034, *P* = 0.0116; time × virus interaction: *F*_(3, 18)_ = 1.9532, *P* = 0.1320). *N* indicates number of mice. Error bars represent s.e.m

To understand the functional role of noncanonical AMPARs inserted during nonfamiliar conspecific exposure, we infused the GluA2-lacking AMPAR blocker NASPM into the VTA starting from the second day of interaction with either social or object stimuli (Fig. 7a, b). NASPM infused mice reduced the interaction with a conspecific stimulus upon repeated exposure (Fig. 7c); by contrast, the infusions did not alter long-term habituation to an object (Fig. 7d), interaction in the home cage between two familiar mice or distance moved in an open field (Supplementary Fig. 7c–e). To further understand the impact of GluA2-lacking AMPARs at VTA DA neuron inputs on conspecific repeated exposure, we promoted the insertion of GluA2-lacking AMPARs via blue-light illumination of ChR2 or eYFP expressing VTA DA neurons^18^ of DAT-Cre mice (VTA::DA^ChR2^: AAV5-Ef1α-DIO-ChR2(H134R)-eYFP, VTA::DA^eYFP^: AAV5-Ef1α-DIO-eYFP). DA neuron stimulation consisted in 15-minute long ChR2-mediated bursts of action potentials^18^ delivered the day before each conspecific exposure (Fig. 7e, f). This noncontingent burst activation increased RI in photocurrent positive neurons (I_ChR2_^+^; Fig. 7g) and blocked habituation to social stimuli (Fig. 7h). Altogether, these data indicate that GluA2-lacking AMPARs might represent a synaptic signature of conspecific saliency and, once inserted, their activity counteracts habituation.

### Nlgn3 loss-of-function impairs novelty-induced plasticity

**Fig. 8:**
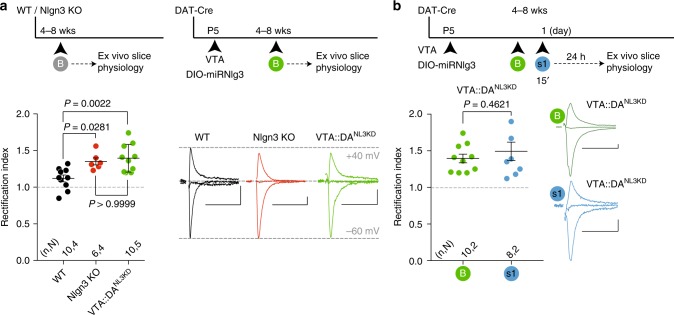
Aberrant increase of GluA2-lacking AMPARs in *Nlgn3*-deficient VTA DA neurons. **a** Top: experimental paradigm. Bottom: scatter plot of rectification index and example traces of AMPAR-EPSCs (−60, 0, and 40 mV) measured from adolescent WT, Nlgn3^KO^ and VTA::DA^NL3KD^. One-way ANOVA (*F*_(2, 23)_ = 8.363, *P* = 0.0019) followed by Bonferroni post hoc test. **b** Top: experimental paradigm. Bottom: scatter plot of rectification index and example traces of AMPAR-EPSCs (−60, 0, and 40 mV) measured from VTA::DA^NLKD^ mice at baseline (B) or 24 h after 15 min exposure to a nonfamiliar conspecific (s1). Unpaired *t* test (*t*_*(*16)_ = 0.7536). *n*, *N* indicates number of cells and mice respectively. Scale bars: 20 msec, 20 pA. Error bars represent s.e.m

*Nlgn3* has been implicated in the regulation of AMPARs at glutamatergic synapses^39^. We therefore hypothesized that defects in DA neuron synaptic function could represent the mechanism underlying the aberrant habituation to familiar conspecifics and response to nonfamiliar conspecifics in VTA::DA^NL3KD^ mice. We explored glutamate receptor function in VTA DA neurons of global *Nlgn3*^*KO*^ and conditional VTA::DA^NL3KD^ mice. Notably, we observed increased RI of AMPAR-mediated currents indicating the aberrant presence of GluA2-lacking AMPARs at excitatory inputs onto VTA DA neurons in both *Nlgn3* loss-of-function models (Fig. 8a). Given the abnormal elevation of GluA2-lacking AMPARs in naïve VTA::DA^NL3KD^ mice, we hypothesized that in these mice synaptic plasticity induced by exposure to nonfamiliar conspecifics might be occluded. Indeed, GluA2-lacking AMPARs in VTA DA neurons were not further increased 24 h after nonfamiliar social stimulus exposure in VTA::DA^NL3KD^ mice (Fig. 8b). Thus, aberrant plasticity of GluA2-lacking AMPARs in VTA DA neurons is associated with an impaired response to a social novel stimulus.

## Discussion

In this study, we establish that intact VTA DA neuron excitability is necessary for (1) the exploration of nonfamiliar social stimuli, (2) the preference for nonfamiliar *versus* familiar conspecifics, and (3) the acquisition of nonfamiliar conspecific-induced contextual associations. Novel stimuli, independent of their nature, leave a plasticity trace at glutamatergic synapses in the VTA, which persists upon repeated exposure to social stimuli and supports sustained conspecific interactions. We use a deletion of the ASD-associated gene *Nlgn3* and demonstrate that global *Nlgn3* knock-out results in an impaired habituation and an aberrant exploration of nonfamiliar conspecifics. Furthermore, selective inactivation of *Nlgn3* in VTA DA neurons disrupts novelty-induced plasticity at glutamatergic synapses in the VTA, alters exploration of nonfamiliar conspecific, and the reinforcing properties of conspecific interactions while having no detectable effect on motor behaviors or olfaction.

Global loss of *Nlgn3* is also accompanied by a broad spectrum of additional phenotypes, including changes in olfaction and in motor-related behaviors^38–41^. Thus, the origin of social behavior alterations in these mice was unclear. Previous studies explored phenotypes in mice carrying a point mutation in *Nlgn3* that reduces (but does not abolish) *Nlgn3* expression and has been observed in 2 patients from one family^43^. For this model, it was concluded that behavioral phenotypes are significantly dependent on the genetic context with significant phenotypes reported for some genetic backgrounds but not others^44–46^. Our study demonstrates that VTA DA neuron specific *Nlgn3* loss of function is sufficient to recapitulate sociability deficits reported in global KO mice.

Although several studies have provided instrumental information about the neuronal circuits, within the reward system, that control social behavior in rodents^13,47–49^, the synaptic adaptations occurring at VTA DA neurons during interactions with nonfamiliar conspecifics remained largely unknown. Here, we show that while reduced excitability or conditional suppression of *Nlgn3* in VTA DA neurons affect the exploration of nonfamiliar conspecifics, they both fail to modify responses to novel objects, presumably because of the higher intrinsic salience of social stimuli. Consistent with this hypothesis, we observe that while both nonfamiliar object and conspecific exploration trigger the insertion of GluA2-lacking AMPARs, only the repeated exposure to the same nonfamiliar mouse results in the maintenance of GluA2-lacking AMPARs at glutamatergic synapses of VTA DA neurons. Therefore, we hypothesize that while the insertion of non-canonical AMPARs reflects the novelty associated to the stimulus, their persistence signals the higher salience of the conspecific over the object stimulus. The insertion and the expression of noncanonical AMPARs has been previously associated with nonsocial and highly salient experiences, such as cocaine exposure^17^. However, a causal relationship between behavioral responses to salient stimuli and GluA2-lacking AMPAR expression at VTA DA neurons has not been reported. Here, we show that the insertion of noncanonical AMPARs at VTA DA neurons contributes to behavioral responses to social stimuli suggesting that these receptors could also represent a functionally-relevant synaptic signature responsible for the behavioral responses associated with other salient stimuli.

Changes in AMPA/NMDA ratio occur in response to both rewarding and aversive processes^50^, and synaptic strengthening is transiently expressed and necessary for associative learning^15^. Consistent with previous findings^16^, we report an increased AMPA/NMDA ratio at VTA DA neuron excitatory inputs in response to social interaction, which is transiently expressed upon repeated exposure to nonfamiliar conspecifics, but not object stimuli. However, although the increased synaptic strength might represent an additional signature related to the saliency of social interaction, its role in habituation processing and, possibly contextual learning, warrants further investigation.

In recent years, accumulating evidences indicate that synaptic adaptations associated to reward and aversion occur at projection-specific subclasses of VTA DA neurons^51,52^. An anatomic-functional segregation of reward circuitry is also emerging in respect of social behavior: while VTA DA neurons projecting to the nucleus accumbens (NAc), but not prefrontal cortex (PFC), promote conspecific interaction^13^, DA neuron projections to the interpenduncular nucleus control familiarity signaling^47^. Thus, the specific synaptic signatures observed in response to nonfamiliar conspecific exposure might also occur in dedicated VTA circuits. At the same time, given the intrinsic diversity of sensory and emotional information provided by social vs. inanimate stimuli, it is conceivable that synaptic plasticity occurs at specific inputs to defined subclasses of VTA DA neurons. Additional investigations of synaptic properties of defined inputs to projection-specific DA neuron subclasses is needed to further understand the circuits and the synaptic mechanisms underlying both novelty and saliency processing associated with conspecific and inanimate stimuli.

Altered social interactions and communication are defining aspects of the autism phenotype. However, such alterations may arise from a plethora of neuronal processing defects, ranging from alterations in perception, sensory processing, multisensory integration, or positive and negative valence assigned to conspecific stimuli^23^. In this work, we specifically explore neuronal circuitry relevant for the exploration to and the preference for nonfamiliar conspecifics. We chose this domain, as studies in children with ASD demonstrated altered habituation and responses to novel stimuli^7,53^. Notably, in toddlers, a slowed habituation to faces but normal habituation to repeatedly viewed objects has been reported to coincide with more severe ASD symptoms^54^. Several rodent models of ASD exhibit altered social novelty responses^24,55^, and such alterations have been suggested to reflect changes in social memory or discrimination. However, brain areas and circuit elements contributing to these changes in habituation and social novelty responses in mice and humans are largely unknown. Our rodent work not only highlights a contribution of VTA DA neurons to this process but also takes steps toward identification of the synaptic basis of social novelty responses and habituation. Considering the complexity of ASD behavioral dysfunctions, we propose that fractionating the autism phenotype according to specific behavioral domains based on neuronal circuit elements will provide a productive stratification criterion for patient populations. Thus, we speculate that in a subpopulation of individuals with ASD alterations in VTA DA function might contribute to the social interaction phenotype whereas in other subgroups of patients alterations in social interaction may arise for different reasons. A prediction from this hypothesis is that stratification of patient populations based on an assessment of novelty responses, habituation, and social reward may help to identify subgroups of patients that would particularly benefit from interventions targeting function and plasticity of the VTA-DA circuit elements.

## Methods

### Animals

The study was conducted with WT and transgenic mice in C57BL/6J background. WT mice were obtained from Charles River. For DA neuron-specific manipulations DAT-iresCre (*Slc6a3*^*tm1.1(cre)Bkmn*^)^56^ and DAT-Cre BAC transgenic mice^57^ were employed. *Nlgn3*^*KO*^ mice were previously described^37^. Male and female mice were housed in groups (weaning at P21 – P23) under a 12 h light – dark cycle (7:00 a.m.–7:00 p.m.). All physiology and behavior experiments were performed during the light cycle. For *Nlgn3*^*KO*^ and WT mice, multiple behavioral tests were performed with the same group of animals, with a minimum of 3 days in-between tests. VTA::DA^NL3KD^ and VTA::GFP participated in one behavioral test prior to the start of social CPP. A minimum of two independent cohorts were used for the behavioral experiments. Embryos for cortical cultures were obtained from NMRI mice (Janvier). All the procedures performed at UNIGE and Biozentrum complied with the Swiss National Institutional Guidelines on Animal Experimentation and were approved by the respective Swiss Cantonal Veterinary Office Committees for Animal Experimentation.

### Surgery

Injections of rAAV5-hSyn-DIO-hM4D(Gi)-mCherry and rAAV5-hSyn-DIO-mCherry were performed in DAT-Cre mice at 4–7 weeks. For additional information on chemogenetic viral vectors, see Supplementary Methods. Mice were anesthetized with a mixture of oxygen (1 L/min) and isoflurane 3% (Baxter AG, Vienna, Austria) and placed in a stereotactic frame (Angle One; Leica, Germany). The skin was shaved, locally anesthetized with 40–50 µL lidocaine 0.5% and disinfected. Bilateral craniotomy (1 mm in diameter) was then performed over the VTA at following stereotactic coordinates: ML ± 0.5 mm, AP −3.2 mm, DV −4.20 ± 0.05 mm from Bregma. The virus was injected via a glass micropipette (Drummond Scientific Company, Broomall, PA) into the VTA at the rate of 100 nl/min for a total volume of 200 nL in each side. The virus was incubated for 3–8 weeks prior to perform the behavioral tasks or electrophysiological recordings.

Injections of purified AAV2-DIO-miRNlgn3-GFP, AAV2-Synaptophysin-GFP and AAV2-DIO-miR-GFP were done at P5–P6 for developmental knockdown and at 4–7 weeks for adult knockdown. For additional information on viral vectors and validation of construct for *Neuroligin 3* downregulation, see Supplementary Methods. Injections were performed under a mixture of oxygen and isoflurane anesthesia (Baxter AG, Vienna, Austria) as previously described. The animals were placed in a stereotaxic frame (Kopf Instrument) and a single craniotomy was made over the VTA at the following stereotaxic coordinates: ML + 0.15 mm, AP + 0.2 mm, DV −4.2 mm from lambda for P5-P6, and for 4–7 weeks: ML ± 0.4 mm, AP -3.2 mm, DV −4.4 mm from Bregma. Injections were made with a 33-G Hamilton needle (Hamilton, 65460-02) for a total volume of 200 nL. Injections sites were confirmed post hoc by immunostaining on VTA. The virus was incubated for 3–4 weeks prior to perform the behavioral tasks or immunostaining. Mice were excluded from the study if the body weight was less than 75% of the mean body weight at the start of behavior trials.

Injections of rAAV5-Ef1α-DIO-hChR2(H134R)-eYFP and rAAV5-Ef1α-DIO-eYFP were performed in DAT-Cre mice at 4–5 weeks. For additional information on viral vectors, see Supplementary Methods. Mice were anesthetized and placed in a stereotactic frame (Angle One; Leica, Germany) as previously described. The skin was shaved, locally anesthetized with 40–50 µL of lidocaine 0.5% and disinfected. Unilateral craniotomy (1 mm in diameter) was then performed to reach the VTA with a 10° angle, at following stereotactic coordinates: ML ± 0.9 mm, AP −3.2 mm, DV −4.20 ± 0.05 mm from Bregma (Paxinos). The virus was injected via a glass micropipette (Drummond Scientific Company, Broomall, PA) into the VTA at the rate of 100 nl/min for a total volume of 500 nL. The virus was incubated for 3–4 weeks and subsequently, mice were implanted with optic fibers above the VTA. The animals were anesthetized, placed in a stereotactic frame, the skin was shaved and a unilateral craniotomy was performed as previously described. The optic fiber was implanted with a 10° angle at the following coordinates: ML ± 0.9 mm, AP −3.2 mm, DV −3.95 ± 0.05 mm from Bregma above the VTA and fixed to the skull with dental acrylic.

Implantations of stainless steel 26-gauge cannula (PlasticsOne, Virginia, USA) were performed on WT mice at 8–10 weeks. Mice were anesthetized and placed in a stereotactic frame as previously described. Unilateral craniotomy (1 mm in diameter) was then performed over the VTA at following stereotactic coordinates: ML ± 0.9 mm, AP −3.2 mm, DV −3.95 ± 0.05 mm from Bregma. The cannula was implanted with a 10° angle, placed above the VTA and fixed on the skull with dental acrylic. Between experiments, the cannula was protected by a removable cap. All animals underwent behavioral experiments 1–2 weeks after surgery.

### Three-chamber test

The three-chambered social preference test was performed in a rectangular Plexiglas arena (60 × 40 × 22 cm) (Ugo Basile, Varese, Italy) divided into three chambers (each 20 × 40 × 22 cm) that communicate by removable doors situated on the walls of the center chamber. Three to eight weeks after virus infusions, VTA::DA^hM4Di^ and VTA::DA^mCherry^ mice were randomly assigned to two batches that received intraperitoneal injections of either saline (vehicle) or Clozapine N-oxide (5 mg kg^−1^; for further details about drug preparation and injection see Supplementary Methods). All injections were done 30 min before starting the experiment. One to two weeks after, the experimental subjects treated first with CNO received vehicle and vice versa, thus performing the task in both conditions. The habituation phase consisted in 10 min of free exploration of the empty arena. Subsequently, the mouse was temporarily kept in the center chamber by closing the removable doors. Two enclosures were placed in the centers of the side chambers. One enclosure was left empty (inanimate object, o1) and the other one contained a nonfamiliar social stimulus (novel juvenile mice C57BL/6J, 3–4 weeks, s1/s3 in vehicle or CNO condition). The doors were then removed and the experimental mouse freely explored the arena and the two enclosures for 10 min. The walls of the enclosures, consisting of vertical metal bars, allowed visual, auditory, olfactory and tactile contact between the experimental mouse and the stimulus mouse. The stimuli mice were habituated to the enclosures during three sessions of 20 min the 3 days before the experiment. The position of the stimuli was randomly assigned and counterbalanced.

The mice were then restrained a second time in the center chamber by closing the removable doors. The enclosures were held in position and a nonfamiliar conspecific (s2/s4 in vehicle or CNO condition) was placed in the empty one. In this phase, the prior nonfamiliar conspecific is considered as familiar (social familiar, s1/s3). The doors were opened and the experimental mouse explored the arena for 10 min, with the two enclosures containing the familiar and the nonfamiliar social stimuli. At the end of the 10 min, the experimental and stimuli mice returned to their home cage.

Every session was video-tracked and recorded using Ethovision XT (Noldus, Wageningen, the Netherlands), which provided the time in the different chambers and the distance moved during the test. An experimenter blind to the treatment of animals also manually scored behavior. The stimulus interaction was scored when the nose of the experimental subject was oriented toward the enclosures at a distance approximately less than 2 cm. The time interaction was used to calculate the Social Novelty Index as: Interaction_nonfamiliar_−Interaction_familiar_. The arena was cleaned with 5% ethanol solution and dried between trials.

### Long-term habituation/nonfamiliar exploration task

An experimental cage similar to the animal’s home cage was used for this task. The bedding was replaced after each trial and water and food were available. During the habituation phase (4 days, day 1–4), all VTA::DA^hM4Di^ experimental mice received an intraperitoneal injection of saline 30 min before the task. The experimental VTA::DA^hM4Di^ mouse was placed in the cage with a nonfamiliar conspecific (novel juvenile mouse, C57Bl/6J, 3–4 weeks old, s1). The animals were let free to explore the cage and to interact with each other for 15 min. At the end of the trial, the experimental and stimulus mice were returned to their homecage. For four consecutive days the experimental mouse was exposed to the same conspecific (s1) and habituated to the environment and the social stimulus. Day 5 consisted in the novelty phase. The VTA::DA^hM4Di^ experimental mice were split in two batches and were injected with either saline or CNO (5 mg kg^−1^) 30 min before the trial. A nonfamiliar conspecific (s2) was placed with the experimental mouse in the cage for 15 min to allow direct interaction. In total, the experimental mice were exposed to two different conspecifics: one social stimulus repeatedly presented from day 1–4 (habituation phase, s1) and a second mouse at day 5 (novelty phase, s2) The same protocol as described above was used for object habituation/nonfamiliar exploration task. The VTA::DA^hM4Di^ experimental mice received injection of saline and were exposed to the same object (der klein kaufman tanner; Germany, o1) from day 1–4 (habituation phase). On day 5 the animals were injected with either saline or CNO (5 mg kg^−1^), and were exposed to a novel object stimulus (novelty phase, o2). VTA::DA^mCherry^ mice underwent the habituation/nonfamiliar exploration task and received an intraperitoneal injection of saline from day 1–4 and CNO (5 mg kg^−1^) on day 5. The social and object habituation/nonfamiliar exploration task performed with *Nlgn3*^*KO*^ and VTA::DA^NL3KD^ was performed as described above. The test was done in a cage similar to the mice home cage containing food and water; the same cage was used for the duration of the trial. Three to four weeks old C57Bl/6J male mice were used as stimulus mice, lego blocks and a small plastic toy were used as object. The animals were left to freely interact with the stimulus mouse or object for 15 min. For four consecutive days the experimental mouse was exposed to the same stimulus (s1 or o1). Day 5 consisted of the novelty phase (s2 or o2). At the end of each trial, the experimental and stimulus mice were returned to their home cage.

During the social habituation/nonfamiliar exploration task, nonaggressive interaction was scored (experimenter blind to genotype and treatment group) when the experimental mouse initiated the action and when the nose of the animal was oriented toward the social stimulus mouse only. During the object habituation/nonfamiliar exploration task, the interaction was scored when the nose of the animal was oriented toward the object stimulus. The time interaction was used to calculate the Novelty Index as: Interaction_Day 5_−Interaction_Day 4_, both for social and object habituation/nonfamiliar exploration task.

The experimental cage was cleaned with 5% ethanol solution and the bedding was changed between sessions.

For the experiments with pharmacological agents, mice were cannulated to allow the infusion of either saline or 1-Naphthylacetyl spermine trihydrochloride (NASPM), directly in the VTA. The habituation task was performed as previously described. NASPM or saline were infused using a Minipump injector (pump Elite 11, Harvard apparatus, US) with 500 nL of saline (2 min of active injection at 250 nL min^−1^ rate, and 1 min at rest), 10 min before each trial. At day 1, mice received saline. From day 2–4 of the habituation phase, mice received either 4 µg of NASPM dissolved in 500 nL of saline or 500 nL of saline only (at 250 nL min^−1^) before each trial. This dose has been previously used to obtain GluA2-lacking AMPARs block in vivo^58^. After at least 1 week, the animals were re-tested to habituation/nonfamiliar exploration task and the pharmacological treatment was counterbalanced. The scoring of the social or object interaction was made as previously described. The experimental cage was cleaned with 5% ethanol solution and the bedding was changed after every session. To assess the cannula placement, experimental subjects were infused using Chicago Sky Blue 6B (1 mg mL^−1^), sacrificed 1–2 h later and transcardially perfused as previously described. For detailed experimental procedures about NASPM infusions during open field and familiar conspecific interaction and VTA DA neuron optogenetic stimulation during habituation/nonfamiliar exploration task, see Supplementary Material and Methods.

### Nonfamiliar, familiar, and novel object CPP

Conditioned place preference experiments for examining reinforcing properties of nonfamiliar conspecific, familiar conspecific or nonfamiliar object interactions were conducted in an apparatus (spatial place preference; BioSEB) consisting of two adjacent chambers (20 × 20 × 25 cm) with dot (black) or stripe (gray) wall patterns, connected by a lateral corridor (7 × 20 × 25 cm) with transparent walls and floor. The dot chamber was always associated to rough floor, while the stripe chamber with smooth floor. The illumination level was uniform between the two chambers and set at 10–13 lux. ANY-Maze behavior tracking software was used to track animal’s movements within the apparatus and to manually score the time spent in nonaggressive interaction with the stimulus.

At day 0, experimental mice (male C57Bl6/J; group-housed; 8–16 weeks) freely explored the CPP apparatus for 15 min to determine Pre-TEST preference for one or the other chamber. After the Pre-TEST, experimental mice returned to their home cage with their cage-mates. The preference score was calculated as time spent in stimulus chamber (US^+^) divided by the sum of the time spent in stimulus chamber (US^+^) and the time spent in the empty chamber. No animals were excluded from the analysis based on preference score and US^+^ pairings were randomly assigned to dot or stripe chamber. At day 0, nonfamiliar conspecific (male C57Bl/6J; single-housed; 3–4 weeks) or familiar stimuli mice (male C57Bl/6J; cohoused with experimental mice during the conditioning) were habituated to the US^+^ chamber for 15–30 min. The novel object stimulus was the same used in the object habituation/nonfamiliar exploration task.

From day 1–4, experimental mice underwent a conditioning schedule consisting of 30 min-long sessions (1 per day). Each session was subdivided in six blocks of 5 min during which the animals alternated between US^+^ and US^−^ chamber, in presence (of either familiar mouse, f1, nonfamiliar mouse, s1 or novel object, o1) or absence (empty) of the stimulus, respectively. Experimental mice were guided through the corridor during the alternations and returned to their home cage with their cage-mates at the end of the conditioning session. Groups were counterbalanced for US^+^/US^−^ sequences and for dot or stripe wall pattern. VTA::DA^hM4Di^ and VTA::DA^mCherry^ mice received an intraperitoneal injection of CNO (5 mg kg^−1^) 30–90 min prior each conditioning session. At day 5, during the Post-TEST, experimental mice freely explored the CPP apparatus, without any stimulus for 15 min and the preference score was measured. The CPP apparatus was cleaned with 1% acetic acid, rinsed with distilled water and dried between each experimental subject.

### Social conditioned place preference

Mice were tested at P30–P45 and were group housed before the test. The test apparatus was a custom-built cage measuring 46 × 24 × 22 cm divided into three chambers. The two outer chambers (23 × 18 × 22 cm) had vertical or horizontal striped pattern on the walls and flooring consisting or black rubber mats with different patterns (stripes *vs* squares). The outer chambers were joined together by a smaller chamber (23 × 10 cm) with white walls and floor with a 7 × 7 cm opening at the base to the outer chambers that can be closed. The cage was cleaned with 70% ethanol between each trial. During the pretrial, mice were left to freely explore the cage for 30 min. After the pretrial, all mice were single housed for the remainder of the test and one chamber was assigned the social chamber and one the isolation chamber. All mice received one social and one isolation condition session (30 min each) per day for 4 days, with a two-day rest between the second and third conditioning day. Mice were socially conditioned for 30 min together with their cage-mates followed by conditioning in the isolation chamber for 30 min. After the fourth conditioning day, mice were tested in a 30 min postconditioning trial. The time spent freely exploring the chambers for 30 min was manually scored by an investigator blinded to the genotype. The preference score was calculated as the time spent in the social chamber divided by the combined time spent in the social and isolation chamber. Animals were excluded by pre-established criteria if they exhibited a strong preference for one chamber (more than 2 reference for one chamber). For detailed description of additional behavioral tasks, see Supplementary Methods.

### Ex vivo electrophysiology

In all, 200–250 µM thick horizontal midbrain slices were prepared from adolescence/early adulthood C57Bl/6J, VTA::DA^hM4Di^, VTA::DA^mCherry^, *Nlgn3* KO and VTA::DA^NL3KD^ mice. Subjects were anesthetized with isoflurane/O_2_ and decapitated. Brains were sliced by using a cutting solution containing: 90.89 mM choline chloride, 24.98 mM glucose, 25 mM NaHCO_3_, 6.98 mM MgCl_2_, 11.85 mM ascorbic acid, 3.09 mM sodium pyruvate, 2.49 mM KCl, 1.25 mM NaH_2_PO_4_, and 0.50 mM CaCl_2_. Brain slices were incubated in cutting solution for 20–30 min at 35°. Subsequently, slices were transferred in artificial cerebrospinal fluid (aCSF) containing: 119 mM NaCl, 2.5 mM KCl, 1.3 mM MgCl_2_, 2.5 mM CaCl_2_, 1.0 mM NaH_2_PO_4_, 26.2 mM NaHCO_3_, and 11 mM glucose, bubbled with 95% O_2_ and 5% CO_2_) at room temperature. Whole-cell voltage clamp or current clamp electrophysiological recordings were conducted at 32°–34° in aCSF (2–3 ml/min, submerged slices). Recording pipette contained the following internal solution: 130 mM CsCl, 4 mM NaCl, 2 mM MgCl_2_, 1.1 mM EGTA, 5 mM HEPES, 2 mM Na_2_ATP, 5 mM sodium creatine phosphate, 0.6 mM Na_3_GTP, 0.1 mM spermine and 5 mM lidocaine N-ethyl bromide. Ex vivo CNO validation experiments were conducted in current-clamp configuration with the following internal solution: 140 mM K-Gluconate, 2 mM MgCl_2_, 5 mM KCl, 0.2 mM EGTA, 10 mM HEPES, 4 mM Na_2_ATP, 0.3 mM Na_3_GTP and 10 mM creatine-phosphate. Putative DA neurons of the VTA were identified accordingly to their position (medially to the medial terminal nucleus of the accessory optic tract), morphology, cell capacitance (>28 pF) and low input resistance at positive potentials. Excitatory postsynaptic currents (EPSCs) were recorded in voltage-clamp configuration, elicited by placing a bipolar electrode rostrolaterally to VTA at 0.1 Hz and isolated by application of the GABA_A_R antagonist picrotoxin (100 µM). Traces were not corrected. Access resistance (10–30 MΩ) was monitored by a hyperpolarizing step of −4 mV at each sweep, every 10 s. Data were excluded when the resistance changed >20%. The AMPA/NMDA ratio was calculated by subtracting to the mixed EPSC (+35 mV), the non-NMDA component isolated by D-APV (50 µM at +35 mV) bath application. The values of the ratio may be underestimated since it was calculated with spermine in the pipette. The rectification index (RI) of AMPARs is the ratio of the chord conductance calculated at negative potential (–60 mV) divided by the chord conductance at positive potential (+40 mV). PPR was measured at −60 mV, with a fixed inter stimulation interval of 50 ms. PPR was calculated by dividing the amplitude of the second EPSC by the amplitude of the first EPSC. To measure the RI from ChR2-expressing VTA DA neurons after in vivo optogenetic stimulation, a 500 ms blue-light pulse was delivered through the microscope objective in voltage-clamp configuration. Neurons were considered photocurrent positive (I_ChR2_^+^) when they responded with a large depolarizing current in response to optical stimulation, while photocurrent negative (I_ChR2_^−^) when they did not. Representative example traces are shown as the average of 10–20 consecutives EPSCs typically obtained at each potential. The synaptic responses were collected with a Multiclamp 700B-amplifier (Axon Instruments, Foster City, CA), filtered at 2.2 kHz, digitized at 5 Hz, and analyzed online using Igor Pro software (Wavemetrics, Lake Oswego, OR). Electrophysiology experiments were performed blind to behavioral or genetic manipulation.

### Statistical analysis

No statistical methods were used to predetermine the number of animals and cells, but suitable sample sizes were estimated based on previous experience and are similar to those generally employed in the field. The animals were randomly assigned to each group at the moment of viral infections or behavioral tests. Statistical analysis was conducted with GraphPad Prism 6 and 7 (San Diego, CA, USA) and MatLab (The Mathwork). Statistical outliers were identified with the ROUT method (*Q* = 1) and excluded from the analysis. The normality of sample distributions was assessed with the Shapiro–Wilk criterion and when violated nonparametrical tests were used. When normally distributed, the data were analyzed with independent *t* test, paired *t* test, while for multiple comparisons one-way ANOVA and repeated measures (RM) ANOVA were used. When normality was violated, the data were analyzed with Mann–Whitney test, Wilcoxon matched-pairs signed rank test, while for multiple comparisons, Kruskal–Wallis or Friedman test were followed by Dunn’s test. For the analysis of variance with two factors (two-way ANOVA, RM two-way ANOVA and RM two-way ANOVA by both factors), normality of sample distribution was assumed, and followed by Bonferroni post hoc test. All the statistical tests adopted were two-sided. When comparing two samples distributions similarity of variances was assumed, therefore no corrections were adopted. For social behavior experiments, the outlier analysis was conducted on manually scored nonaggressive social interaction. Data are represented as the mean ± s.e.m. and the significance was set at *P* < 0.05. For further information about the statistical analysis of the three-chamber social interaction task and a summary of the statistical tests adopted for each dataset, see Supplementary Methods and Supplementary Table 1.

### Data availability

The data supporting this study are available upon request to the corresponding author.

## Electronic supplementary material

Supplementary Information
