# Supplementary material for: Role of VTA dopamine neurons and neuroligin 3 in sociability traits related to nonfamiliar conspecific interaction
Source: Nat Commun. 2018 Aug 9;9:3173. doi: 10.1038/s41467-018-05382-3 (PMC6085391; doi:10.1038/s41467-018-05382-3)
Supplement: Supplementary file 1 — Supplementary Information [file 41467_2018_5382_MOESM1_ESM.pdf]

Supplemetary information

Role of VTA dopamine neurons and Neuroligin3 in sociability traits related to non-familiar conspecific interaction.

Bariselli, Hornberg, Prevost-Solie et al.,

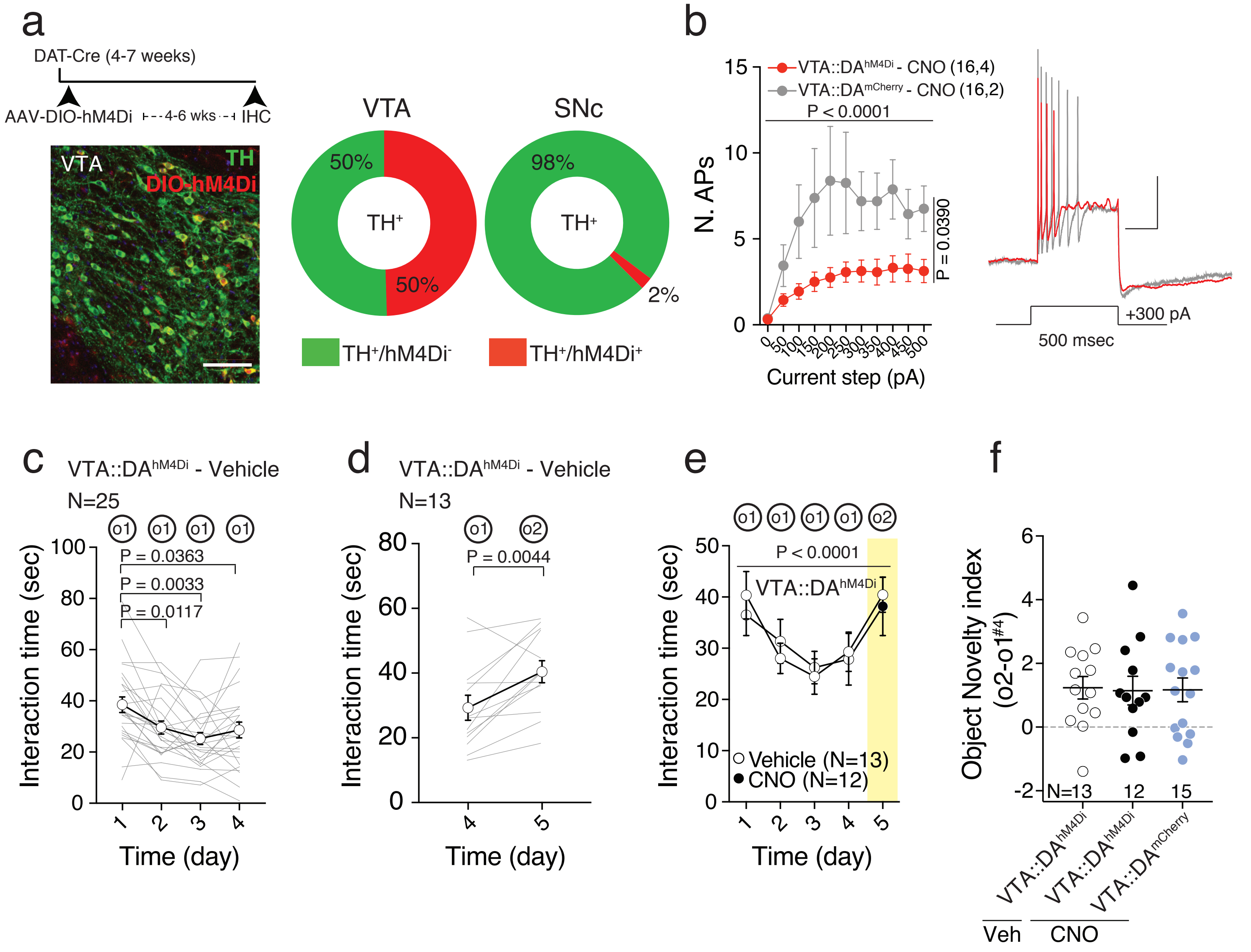

Supplementary Figure1

Supplementary figures

Supplementary Figure 1. VTA DA neuron excitability controls non-familiar conspecific exploration.

(a) Top left: experimental paradigm. Bottom left: representative image of VTA DA neurons infected with AAV5-DIO-hM4Di-mCherry and stained against TH enzyme. Scale bar: 100 µm. Right: quantification of viral infection of TH^+ neurons of VTA and Substantia Nigra pars compacta (SNc). **(b)** Left: graph representing the number of action potentials fired in response to 500 msec of increasing steps of current amplitude for $VTA::DA^{hM4Di}$ and $VTA::DA^{mCherry}$ neurons in presence of CNO (10 μM). RM two-way ANOVA (current main effect: $F_{(10, 300)}$ = 6.513, P < 0.0001; virus main effect: $F_{(1, 30)}$ = 4.66, P = 0.039; current X virus interaction: $F_{(10, 300)}$ = 1.527, P = 0.1287). Right: example traces of action potentials measured in current-clamp for $VTA::DA^{hM4Di}$ and $VTA::DA^{mCherry}$ neurons in CNO. Scale bar: 200 msec, 20 mV. **(c)** Time course of time interaction for $VTA::DA^{hM4Di}$ mice treated with vehicle. RM one-way ANOVA ($F_{(2.31, 55.44)}$ = 6.925, P = 0.0013) followed by Bonferroni post-hoc test for planned multiple comparisons. **(d)** Graph reporting the time interaction at day 4 with o1 and at day 5 with o2 for $VTA::DA^{hM4Di}$ mice treated with vehicle. Paired t-test ($t_{(12)}$ = 3.492). **(e)** Time interaction over days during habituation/non-familiar exploration task for vehicle and CNO treated $VTA::DA^{hM4Di}$ mice (o1 and o2 are object non-familiar stimuli presented at day 1-4 and day 5). RM two-way ANOVA (time main effect: $F_{(4, 92)}$ = 8.314, P < 0.0001; drug main effect: $F_{(1, 23)}$ = 0.01301, P = 0.9102; time X drug interaction: $F_{(4, 92)}$ = 0.4707, P = 0.7571). **(f)** Object novelty index calculated from $VTA::DA^{hM4Di}$ treated with vehicle, $VTA::DA^{hM4Di}$ and $VTA::DA^{mCherry}$ treated with CNO. One-way ANOVA ($F_{(2,37)}$ = 0.0144, P = 0.9857).

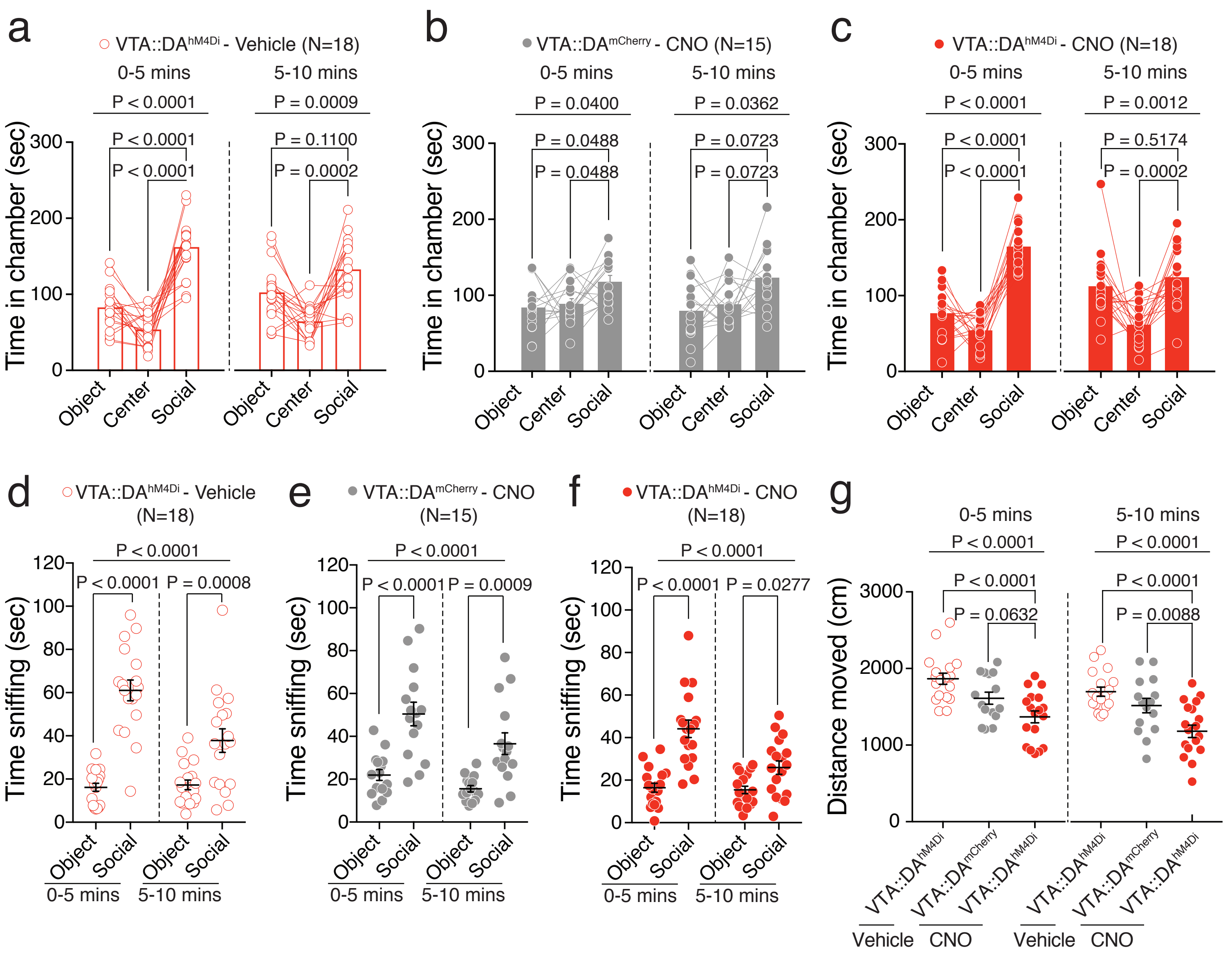

Supplementary Figure 2

Supplementary Figure 2. Decreasing VTA DA neuron excitability in the 3-chamber task preserves preference for non-familiar conspecific over object.

(**a**) Time in chamber for vehicle treated VTA::DA^{hM4Di} mice. RM one-way ANOVA (chamber main effect: $F_{(1.519, 25.82)} = 42.35$, $P < 0.0001$, first 5 mins; chamber main effect: $F_{(1.426, 24.25)} = 11.63$, $P = 0.0009$, last 5 mins) followed by Holm-Sidak post-hoc test. (**b**) Time in different stimuli chamber of CNO treated VTA::DA^{mCherry} mice. RM one-way ANOVA (chamber main effect: $F_{(1.857, 26)} = 3.748$, $P = 0.0400$, first 5 mins; chamber main effect: $F_{(1.725, 24.15)} = 4.026$, $P = 0.0362$, last 5 mins) followed by Holm-Sidak post-hoc test. (**c**) Time in different chamber for CNO treated VTA::DA^{hM4Di} mice. RM one-way ANOVA (chamber main effect: $F_{(1.777, 30.21)} = 56.17$, $P < 0.0001$, first 5 mins; chamber main effect: $F_{(1.653, 28.11)} = 9.611$, $P = 0.0012$, last 5 mins) followed by Holm-Sidak post-hoc test. (**d**) Time sniffing toward non-familiar conspecific or object stimuli for vehicle treated VTA::DA^{hM4Di} mice. RM two-way ANOVA (stimulus main effect: $F_{(1, 34)} = 43.28$, $P < 0.0001$; time main effect: $F_{(1, 34)} = 21.54$, $P < 0.0001$; time X stimulus interaction: $F_{(1, 34)} = 25.97$, $P < 0.0001$) followed by Bonferroni post-hoc test. (**e**) Time sniffing toward social or object stimuli for CNO treated VTA::DA^{mCherry} mice. RM two-way ANOVA (stimulus main effect: $F_{(1,28)} = 23.65$, $P < 0.0001$; time main effect: $F_{(1,28)} = 17.22$, $P = 0.0003$; time X stimulus interaction: $F_{(1,28)} = 2.311$, $P = 0.1397$) followed by Bonferroni post-hoc test. (**f**) Time sniffing toward non-familiar conspecific or object stimuli for CNO treated VTA::DA^{hM4Di} mice. RM two-way ANOVA (stimulus main effect: $F_{(1,34)} = 34.13$, $P < 0.0001$; time main effect: $F_{(1,34)} = 14.14$, $P = 0.0006$; time X stimulus interaction: $F_{(1,34)} = 11.22$, $P = 0.0020$) followed by Bonferroni post-hoc test. (**g**) Distance moved, binned in 5 mins, for VTA::DA^{hM4Di} vehicle and CNO treated mice, and VTA::DA^{mCherry} CNO treated mice. One-way ANOVA (group main effect: $F_{(2, 48)} = 11.26$, $P < 0.0001$, first 5 mins; group main effect: $F_{(2, 48)} = 12.03$, $P < 0.0001$, last 5 mins) followed by Bonferroni post-hoc test for planned comparisons. N indicates number of mice. Error bars report s.e.m.

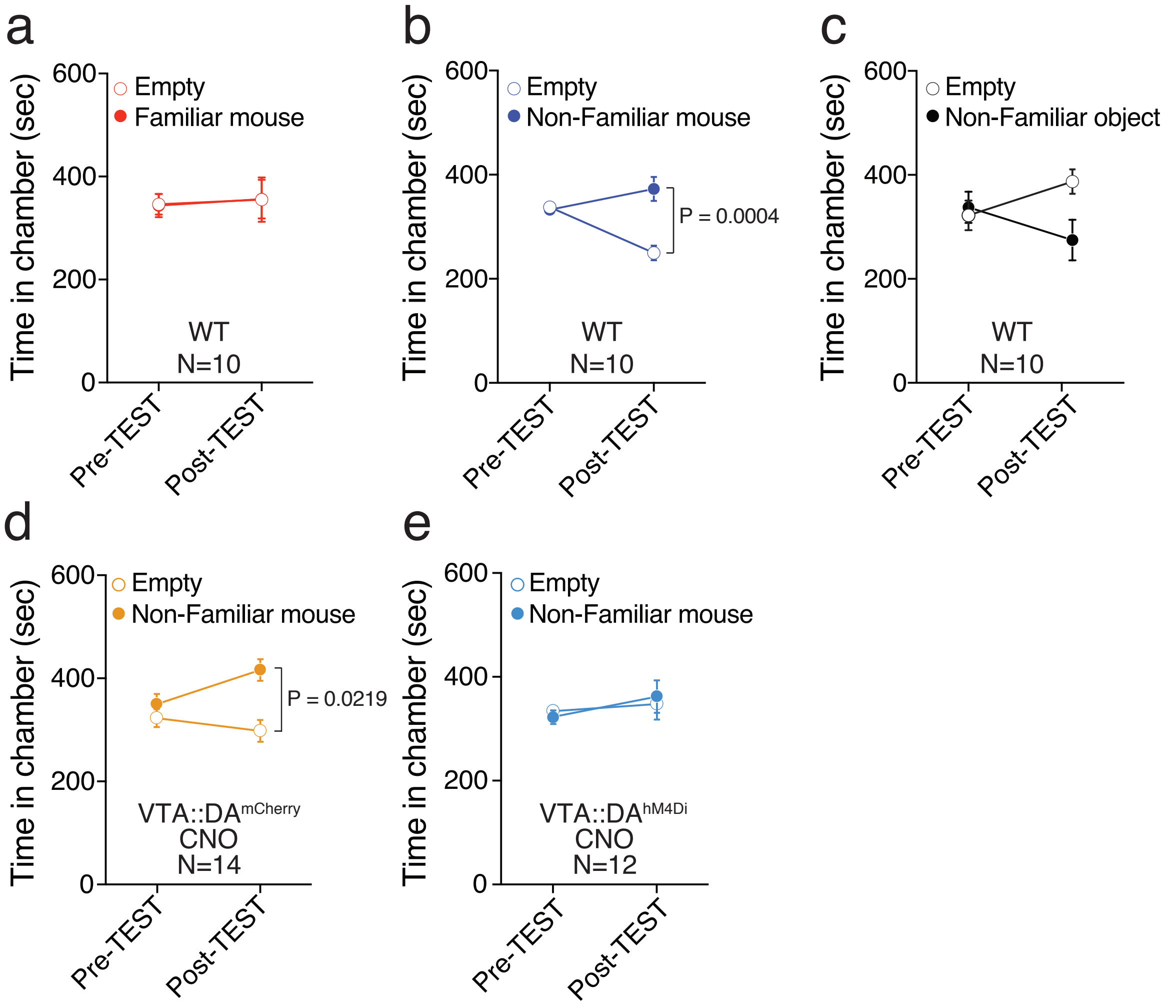

Supplementary Figure 3

Supplementary Figure 3. Time of chamber exploration during CPP.

(**a**) Graph representing the time spent in either empty or familiar mouse paired chamber during the Pre- and the Post-TEST. RM two-way ANOVA by both factors (time main effect: $F_{(1, 9)}$ = 1.499, P = 0.2519; chamber main effect: $F_{(1, 9)}$ = 0.0002, P = 0.9885; time X chamber interaction: $F_{(1, 9)}$ = 0.0033, P = 0.9557). (**b**) Graph representing the time spent in either empty or non-familiar mouse paired chamber during the Pre- and the Post-TEST. RM two-way ANOVA by both factors (time main effect: $F_{(1, 9)}$ = 11.45, P = 0.0081; chamber main effect: $F_{(1, 9)}$ = 5.313, P = 0.0466; time X chamber interaction: $F_{(1, 9)}$ = 19.4, P = 0.0017) followed by Bonferroni post-hoc test. (**c**) Graph representing the time spent in either empty or non-familiar object paired chamber during the Pre- and the Post-TEST. RM two-way by both factors (time main effect: $F_{(1, 9)}$ = 0.0098, P = 0.9232; chamber main effect: $F_{(1, 9)}$ = 0.9655, P = 0.3515; time X chamber interaction: $F_{(1, 9)}$ = 3.912, P = 0.0793). (**d**) Graph representing the time spent in either empty or non-familiar conspecific stimulus paired chamber during the Pre- and the Post-TEST for VTA::DA^{mCherry} animals treated with CNO. RM two-way ANOVA by both factors (time main effect: $F_{(1, 13)}$ = 0.7315, P = 0.4002; chamber main effect: $F_{(1, 13)}$ = 26.9361, P < 0.0001; time X chamber interaction: $F_{(1, 13)}$ = 3.5527, P = 0.0707) followed by Bonferroni post-hoc test. (**e**) Graph representing the time spent in either empty or non-familiar conspecific stimulus paired chamber during the Pre- and the Post-TEST for VTA::DA^{hM4Di} animals treated with CNO. RM two-way ANOVA by both factors (time main effect: $F_{(1, 11)}$ = 1.7620, P = 0.1980; chamber main effect: $F_{(1, 11)}$ = 0.0040, P = 0.9503; time X chamber interaction: $F_{(1, 11)}$ = 0.4080, P = 0.5296). N indicates number of mice. Error bars report s.e.m.

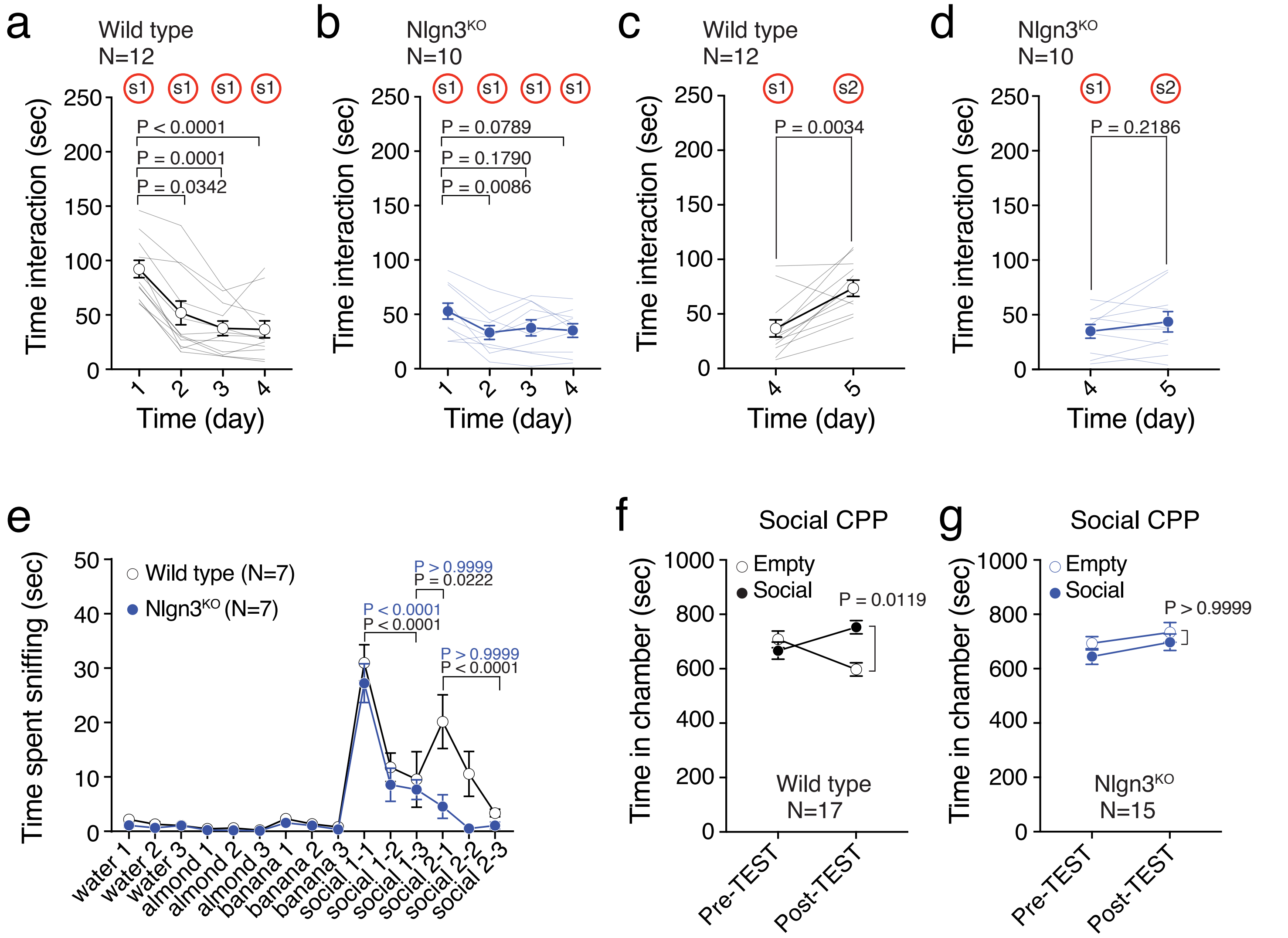

Supplementary Figure 4

Supplementary Figure 4. Global knockdown of *Nlgn3* alters social exploration and the reinforcing properties of conspecific interaction.

(**a-b**) Time spent interacting with the stimulus mouse during day 1-4 of the non-familiar conspecific exploration test plotted for (**a**) WT (Friedman test ($x^2_{(4)}$ = 26.8, P < 0.0001) followed by Dunn's post-hoc test for planned multiple comparisons) and (**b**) *Nlgn3^{KO}* mice (RM ANOVA ($F_{(2.054,\ 18.48)}$ = 5.179, P = 0.0158) followed by Bonferroni post-hoc test for planned multiple comparisons). (**c-d**) Time interacting with s1 on day 4 and s2 on day 5 plotted for (**c**) WT (Wilcoxon test (W = 70) and (**d**) *Nlgn3^{KO}* mice (Paired t-test ($t_{(9)}$ = 1.323). (**e**) Mean time spent sniffing the odor plotted for WT and *Nlgn3^{KO}* mice. RM two-way ANOVA within genotype to evaluate habituation (P-values displayed in graph) and between genotype to evaluate differences in response (social 2-1: WT vs *Nlgn3^{KO}* P<0.0001, social 2-2: WT vs *Nlgn3^{KO}* P=0.0064) (odor main effect: $F_{(14,\ 168)}$ = 30.42, P < 0.0001; genotype main effect: $F_{(1,\ 12)}$ =15.42, P = 0.0020; odor X genotype interaction: $F_{(14,\ 168)}$ = 2.45, P = 0.0036) followed by Bonferroni's post-hoc test. (**f-g**) Time spent in empty or conspecific chamber during the social CPP Pre- and Post-TEST plotted for (**f**) WT (Two-way ANOVA with repeated measures for both factors (time main effect: $F_{(1,16)}$ = 1.361, P = 0.2604; chamber main effect: $F_{(1,16)}$ = 2.22, P = 0.1557; time X chamber interaction: $F_{(1,16)}$ = 8.07, P = 0.0118) followed by Bonferroni post-hoc test) and (**g**) *Nlgn3^{KO}* (Two-way ANOVA with repeated measures for both factors time main effect: $F_{(1,14)}$ = 6.992, P = 0.0192; chamber main effect: $F_{(1,14)}$ = 1.233, P = 0.2855; time X chamber interaction: $F_{(1,14)}$ = 0.0225, P = 0.8827) followed by Bonferroni post-hoc test). N indicates number of mice. Error bars report s.e.m.

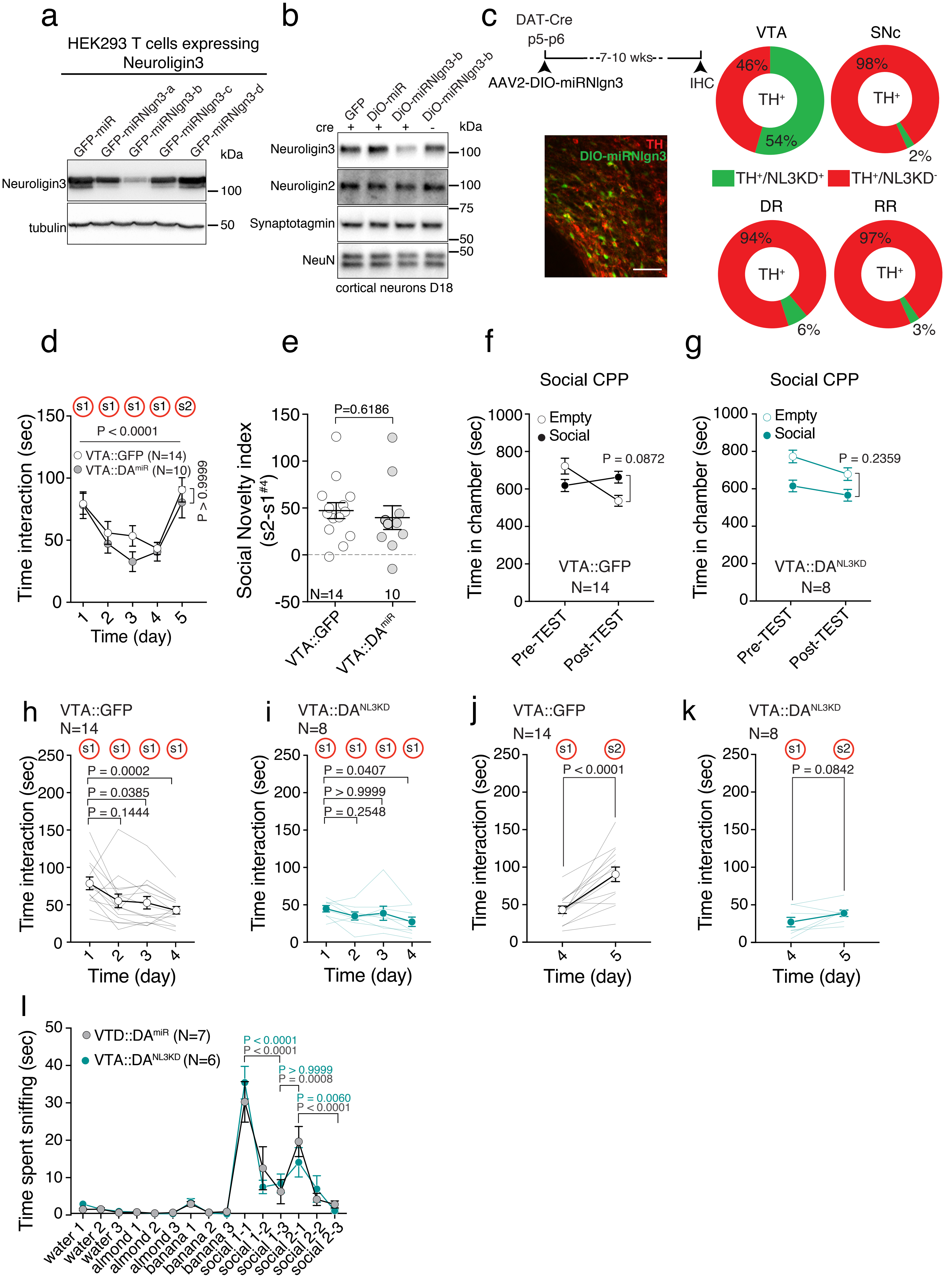

Supplementary Figure 5

Supplementary Figure 5. ***Nlgn3*** **in VTA DA neurons is required for exploration of non-familiar conspecific.**

(**a-b**) Representative western blots showing efficiency of *Nlgn3* miR knockdown in (**a**) HEK293T cells and (**b**) cortical neurons. (**c**) Top: experimental paradigm. Left: VTA DA neurons infected with AAV2-DIO-miR^{Nlgn3}. Scale bar 100 μm. Right: Quantification of viral infection in TH^{+} neurons in VTA, SNc, dorsal raphe nucleus (DR) and retrorubral area (RR). Mean from 8 animals. (**d-e**) DA*T-Cre* mice VTA-injected with a non-targeting, cre-dependent miR (VTA::DA^{miR}) compared to the VTA::GFP mice in the habituation/non-familiar conspecific exploration task. (**d**) Time interaction over days. RM two-way ANOVA (time main effect: $F_{(4, 88)}$ = 19.42, P < 0.0001, virus main effect: $F_{(1, 22)}$ = 0.8246, P = 0.3737; time virus interaction: $F_{(4, 88)}$ = 0.6914, P = 0.5998). (**e**) Social novelty index. Unpaired t-test ($t_{(22)}$ = 0.505)). (**f-g**) Time in chamber for social CPP task (**f**) VTA::GFP and (**g**) VTA::DA^{NL3KD} mice. RM two-way ANOVA for both factors. VTA::GFP: Time main effect: $F_{(1,13)}$ = 9.285, P = 0.0094; chamber main effect: $F_{(1,13)}$ = 0.06717, P = 0.7996; time X chamber interaction: $F_{(1,13)}$ = 8.26, P = 0.0130. VTA::DA^{NL3KD}: Time main effect: $F_{(1,7)}$ = 8.405, P = 0.0230; chamber main effect: $F_{(1,7)}$ = 14.49, P = 0.0067; time X chamber interaction: $F_{(1,7)}$ = 0.2515, P = 0.6314. (**h-i**) Time interaction with conspecific during habituation for (**h**) VTA::GFP (Friedman test ($x^2_{(4)}$ = 16.8, P = 0.0008) followed by Dunn's post-hoc test for planned multiple comparisons) (**i**) and VTA::DA^{NL3KD} (RM ANOVA ($F_{(1.48, 10.36)}$ = 3.098, P = 0.0978) followed by Bonferroni post-hoc test for planned multiple comparisons). (**j-k**) Time interaction with s1 on day 4 and s2 on day 5 for (**j**) VTA::GFP (Paired t-test ($t_{(13)}$ = 5.537) and (**k**) VTA::DA^{NL3KD} (Paired t-test ($t_{(7)}$ = 2.011). (**l**) Mean time sniffing the odors. RM two-way ANOVA within (P-values displayed in graph) and between virus injection (odor main effect: $F_{(14, 154)}$ = 34.26, P < 0.0001; virus main effect: $F_{(1, 11)}$ =0.0026, P = 0.9602; odor X genotype interaction: $F_{(14, 154)}$ = 0.7935, P = 0.6752). RM two-way ANOVAs were followed by Bonferroni post-hoc test. N numbers indicate mice. Error bars show s.e.m.

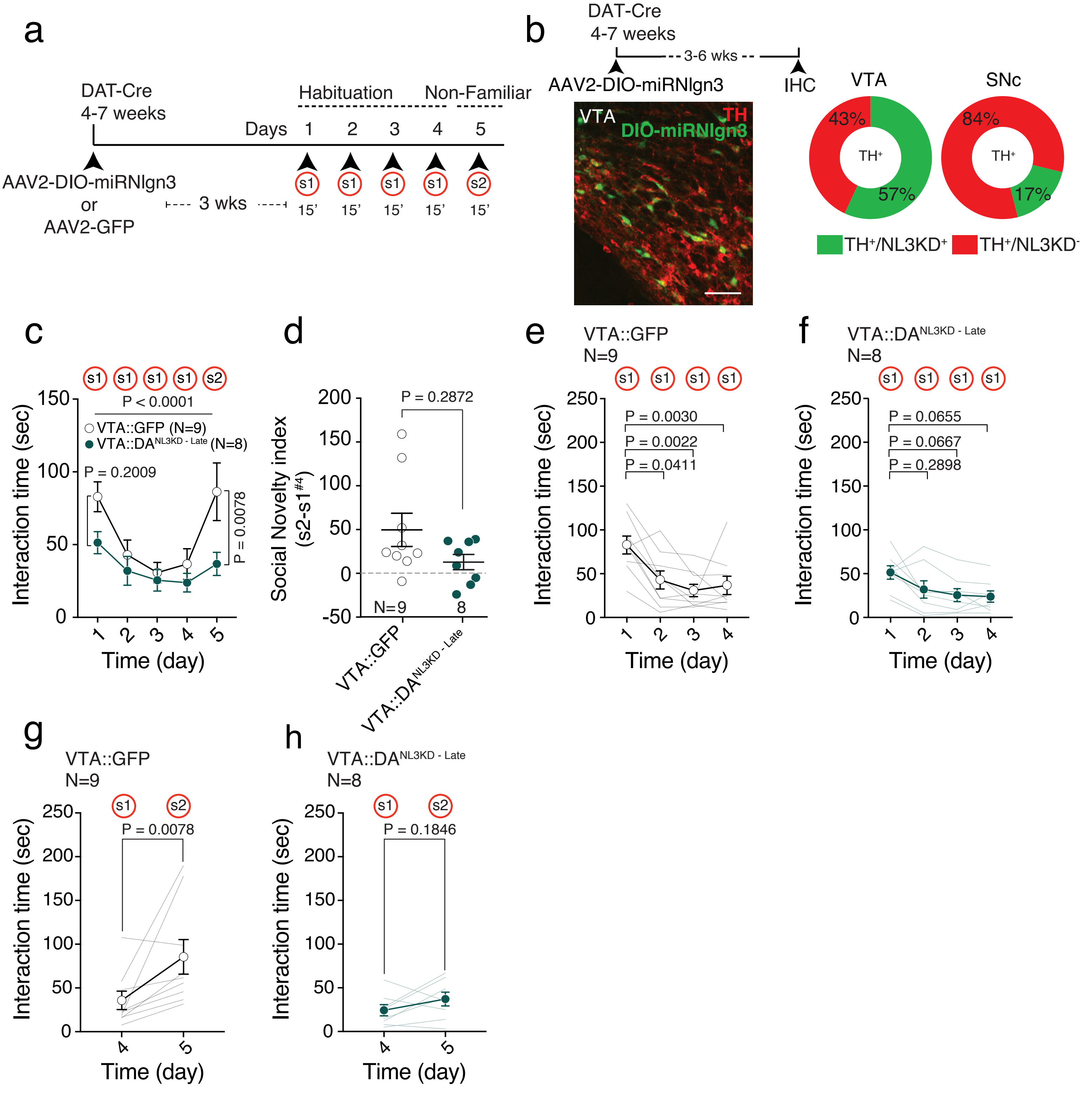

Supplementary Figure 6

Supplementary Figure 6. Adult *Nlgn3* knockdown in VTA DA neurons alters response for non-familiar conspecific.

(**a**) Experimental schematics in adult VTA-injected mice. (**b**) Top: experimental timeline for in vivo knock-down of NL3 expression in adult VTA-DA neurons. Scale bar: 100 µm. Left: Representative image of confocal section stained with anti-TH antibodies and visualizing GFP fluorescence driven from the AAV2-DIO-miR^{Nlgn3}. Right: Quantification of viral infection in TH-positive neurons in the VTA and SNc of DA*T-Cre* mice. TH^{+}/ $NL3KD^{+}$: double-positive neurons; TH^{+}/$NL3KD^{-}$: TH-positive GFP-negative neurons. Percentage represent mean from 8 animals. (**c**) Mean social interaction plotted for VTA::GFP and VTA::DA^{NL3KD} adult-injected mice. RM two-way ANOVA (time main effect: $F_{(4, 60)} = 10.18$, $P < 0.0001$, virus main effect: $F_{(1, 15)} = 3.91$, $P = 0.0667$; time X virus interaction: $F_{(4, 60)} = 2.579$, $P = 0.0463$) followed by Bonferroni's post-hoc test. (**d**) Social novelty index for adult-injected VTA::GFP and VTA::DA^{NL3KD} mice. Mann-Whitney U=24.5. (**e-f**) Time spent interacting with the stimulus mouse during day 1-4 of the habituation/non-familiar conspecific exploration test plotted for (**e**) adult-injected VTA::GFP (Friedman test ($x^2_{(4)} = 15.07$, $P = 0.0018$) followed by Dunn's post-hoc test for planned multiple comparisons) (**f**) and adult-injected VTA::DA^{NL3KD} mice. RM ANOVA ($F_{(1.6, 11.2)} = 5.837$, $P = 0.0228$) followed by Bonferroni post-hoc test for planned multiple comparisons. (**g-h**) Time interacting with s1 on day 4 and s2 on day 5 plotted for (**g**) adult-injected VTA::GFP (Wilcoxon (W= 43) and (**h**) adult-injected VTA::DA^{NL3KD} mice (Paired t-test ($t_{(7)} = 1.472$). N numbers indicate mice. Error bars show s.e.m.

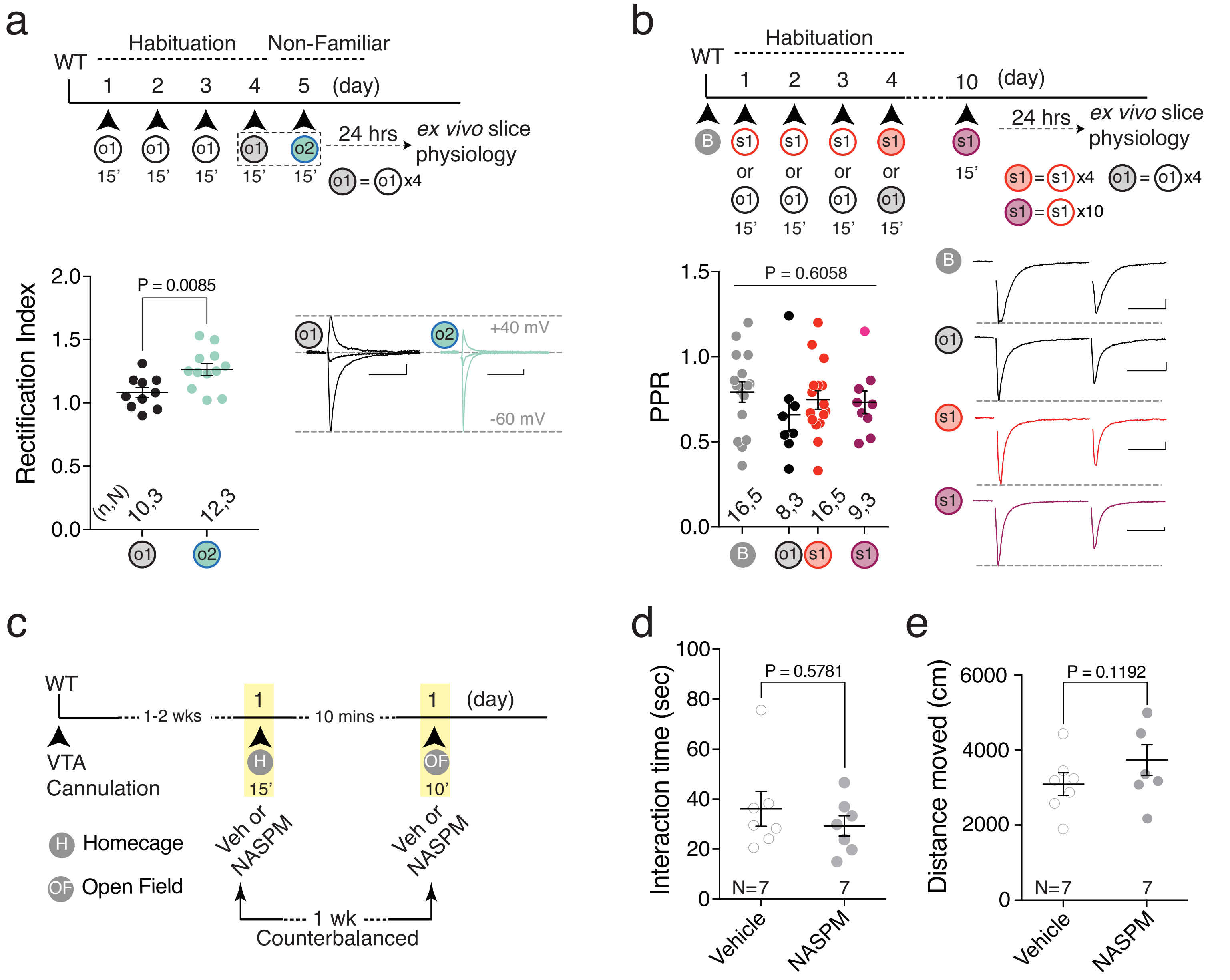

Supplementary Figure 7

Supplementary Figure 7. Additional characterization of synaptic and behavioural parameters during habituation/non-familiar exploration task.

(**a**) Top: experimental paradigm. Bottom: scatter plot and example traces of rectification index measured from VTA DA neurons 24 hours after 4 repeated exposures to the same non-familiar object (o1) or 24 hours after exposure to a non-familiar object (o2) after 4 days exposure to o1. The data reported for o1 are the same used in Figure 6c. Unpaired t-test ($t_{(20)}$ = 2.918). (**b**) Top: experimental paradigm. Bottom: scatter plot and example traces of Paired-Pulse Ratio (PPR, -60 mV) measured 24 hours after 4 repeated exposures to either the same non-familiar object (o1) or the same non-familiar conspecific (s1, red) and 24 hours after 10 repeated exposures to the same non-familiar mouse (s1, purple). One-way ANOVA ($F_{(3, 45)}$ = 0.6058, P = 0.6147). **(c)** Experimental paradigm of familiar conspecific interaction in home cage and distance travelled in open field of vehicle and NASPM VTA-infused mice. Mice received both vehicle and NASPM in each condition with 1 week interval. **(d)** Scatter plot of time interaction measured for 15 minutes in the home cage with familiar conspecific of mice infused with either vehicle or NASPM. Wilcoxon (W= -8). **(e)** Scatter plot of distance travelled in open-field for 10 minutes of mice infused with either vehicle or NASPM. Paired t-test ($t_{(6)}$ = 1.816). Scale bars: 20 msec, 20 pA. n,N indicates number of cells and mice respectively. Error bars show s.e.m.

Supplementary Methods

Drugs and viruses

rAAV5-hSyn-DIO-hM4D(Gi)-mCherry (Titer ≥ 3×10^{12} vg.mL^{-1}, Addgene), rAAV5-hSyn-DIO-mCherry (Titer ≥ 3×10^{12} vg.mL^{-1}, Addgene), rAAV5-Ef1α-DIO-hChR2(H134R)-eYFP (Titer ≥ 4.2×10^{12} vg.mL^{-1}, UNC Vector Core), rAAV5-Ef1α-DIO-eYFP (Titer ≥ 4.2×10^{12} vg.mL^{-1}, UNC Vector Core), pAAV2-Syn-DIO-miRNlgn3-GFP (Titer ≥ $7.2\text{x}10^{9}$ vg.mL^{-1} , miRNlgn3 RNAi BLOCK-iT RNAi Design, Invitrogen), pAAV2-Syn-DIO-miR-GFP (Titer ≥ $8.8\text{x}10^{11}$ vg.mL^{-1} , RNAi BLOCK-iT RNAi Design, Invitrogen), pAAV2-Syn-GFP (Titer ≥ $1.4\text{x}10^{13}$ vg.mL^{-1}), pAAV2-DIO-sh negative-GFP (Titer ≥ $8.8\text{x}10^{11}$ vg.mL^{-1} , RNAi BLOCK-iT RNAi Design, Invitrogen), pAAV2-Syn-iCre (Titer ≥ $2.1\text{x}10^{12}$ vg.mL^{-1}). Clozapine-N-oxyde (BML-NS105, Enzo), 1-Naphthylacetyl spermine trihydrochloride (24897539, Sigma-Aldrich), Picrotoxin (1128, Tocris) and D-APV (0106, Tocris).

Intra-peritoneal injection of saline and Clozapine-N-oxyde (CNO)

The mice were weighted before each experiment and intra-peritoneal (i.p.) injection. The CNO (purchased from Enzo Life Science, Farmingdale, USA) dose was based on previous publications[1] and a concentration of 5 mg kg^{-1} was used for all the experiments. The CNO was diluted in saline to obtain a concentration of 0.5 mg mL^{-1} to inject a reasonable volume of solution. The volume of saline (vehicle) injection was comparable to the volume of CNO solution.

Immunohistochemistry and cell counting

VTA::DA^{hM4Di} infected mice were deeply anesthetized and trans-cardially perfused with PBS 1× followed by 4% paraformaldehyde prepared in PBS 1×. The brain was removed and left for post-fixation at 4 °C in PBS 1×. Coronal VTA slices were cut at 50 μm and washed three times in PBS 1× before incubation with blocking solution containing 0.3% Triton X-100 and 1% goat serum. Slices were incubated with rabbit anti-TH (Abcam ab112, 1:500) at 4 °C overnight and then washed three times in PBS 1× and incubated for 2 hours at room temperature with secondary antibodies, goat anti-rabbit IgG-Alexa 488 (Abcam, 1:500; ab150077). Finally, the slices were washed three times in PBS 1× before being mounted onto microscope slides with Abcam DAPI mounting

medium (Abcam, ab104139). Images were acquired with an LSM-700 confocal microscope.

Cell counting was performed on 50 μm thick VTA slices from 5 VTA::DA^{hM4Di}. For each slice, images from the VTA and SNc were acquired bilaterally along the whole VTA dorso-ventral axis. The TH^{+}, mCherry^{+} and TH^{+}/mCherry^{+} cells were counted from different field of view. The total percentage of cells was calculated by averaging the total number of TH^{+} and TH^{+}/mCherry^{+} of each mouse. The same procedure was performed for the SNc. An immunochemistry was performed for all the mice to assess viral expression. Non-infected animals were excluded from the analysis.

VTA::DA^{NL3KD} mice were perfused as described above. Tissues were sectioned at 35 μm on a cryostat (Microm HM650, Thermo Scientific). Floating sections were kept in PBS 1× before incubation with blocking solution containing 0.5% Triton X-100 in TBS 1x and 10% normal donkey serum. The slices were incubated with sheep anti-TH (Millipor, AB1542, 1:1000) at 4 °C overnight and washed three times in 1x TBS containing 0.5% Triton X-100, followed by incubation for 2 hours at room temperature with a secondary antibody, donkey anti-sheep IgG-Cy3 (Jackson ImmunoResearch, 713-165-147, 1:1000). The sections were washed three times in TBS 1x containing 0.5% Triton X-100 before mounted onto microscope slides with ProLong Gold antifade (Invitrogen, p36930). Images were acquired on a custom-made dual spinning disk microscope (Life Imaging Services GmbH, Basel Switzerland) using 10x and 40x objectives. Images of brain regions expressing DAT; the VTA, SNc, dorsal raphe nucleus (DR) and retrorubral area (RR) were taken bilaterally along the whole dorso-ventral axis and images from at least 3 slices were counted. VTA::DA^{NL3KD} mice were included if a minimum of 20% of cells in the VTA were TH and GFP positive. Total percentage of infected cells was calculated by averaging the percentage obtained for each mouse.

Acute familiar exposure and Open Field with NASPM/saline.

Mice were cannulated (at 8 – 10 weeks of age) and housed two per cage. After recovery (1 – 2 weeks), subjects were infused with either 500 nL of saline or 500 nL of NASPM (4 μ g in 0.5 μ L at 250nL min^{-1}) 10 minutes before the trial. After infusion, mice returned to their home cage and the direct non-aggressive interaction with their cage-mates was immediately scored for 15 consecutive minutes. 10 – 15 minutes after the

social familiar exposure, the cannulated mice were placed in an open field arena for 10 minutes. The apparatus consisted in a 45 cm sided Plexiglas squared arena. After the test, experimental mice returned to their homecage. After 1 week the mice were re-tested and the animals that performed the task under NASPM received saline and *vice versa*. At the end, all the mice underwent both conditions. The familiar social interaction was manually scored as described previously and the open field task was video-tracked (Ethovision, Noldus, Wageningen, the Netherlands) to automatically obtain the distance and the velocity during the session. The arena was cleaned with 5% ethanol solution after every session.

Olfactory habituation/dishabituation test

The olfactory habituation/dishabituation test was performed as previously described[2]. Briefly, mice were individually tested for time spent sniffing cotton tipped swabs suspended from the cage lid. Distilled water, almond flavoring and banana flavoring (McCormick, Hunt Valley, MD; 1:100 dilution) and two different social odors were tested. Social odors were originated from two cages with the same number of male mice with different parental origins maintained for 6 days in the same bedding. Before the test, swabs were wiped in a zig-zag pattern across the bottom surface to collect the olfactory cues. Mice were acclimatized for 30 min with a cotton swab before testing. The order of presentation was: water, water, water, almond, almond, almond, banana, banana, banana, social odor 1, social odor 1, social odor 1, social odor 2, social odor 2, and social odor 2. Each swab was presented for a 2 min period, with a 1min interval between each presentation. Each test session was conducted in a clean mouse cage containing fresh litter. Time spent sniffing the swab was manually scored, the observers were blind of the genotype. Sniffing was scored when the nose was within 2 cm of the cotton swab. Mice were excluded by pre-established criteria if they did not investigate the first social odor (1 WT and 1 $Nlgn3^{KO}$ excluded).

Open field, object recognition task, and marble burying

On day 1, mice were placed individually in the center of a square open field arena (50x50x30 cm) made of grey plastic for 7 minutes. Velocity (cm sec^{-1}) was analyzed using EthoVision10 system (Noldus). The arena was cleaned with 70% ethanol between trials. 24 hours later, mice were placed back in the arena containing two identical

objects (culture flask filled with sand) for a 5-minutes acquisition trial. Object recognition memory was tested 1 hour later during a 5-minutes test trial in the arena containing a familiar and novel object (Lego block). The trial was recorded with a video camera and the time spent investigating was scored manually, the experimenters were blinded to the genotype. Investigation of the object was considered when the mouse nose was sniffing less than a centimeter from or touching the object. The discrimination ratio was calculated as following: $\frac{Time\ spent\ investigating\ novel\ object + familiar\ object}{total\ time\ investigating}$. The arena and objects were cleaned with 70% ethanol between trials.

For the marble-burying test, animals were placed in a standard Type II cage with 5 cm bedding containing 20 identical black marbles distributed equally for 30 minutes. A marble was considered buried if at least 2/3 of the marble was covered.

Cell culture

HEK293T (ATCC) transfected with V5-tagged *Nlgn3* and different NL3 knockdown plasmids (RNAi BLOCK-iT RNAi Design, Invitrogen) were maintained in DMEM supplemented with 10% FBS for 24 hours at 37°C after transfection. The HEK293T cells were tested for mycoplasma contamination using MycoAlert Mycoplasma detection kit (Lonza # LT07-118) and with DAPI/Hoecht staining. Cortical cultures were prepared from E16.5 mouse embryos. Neocortices were dissociated by addition of papain (130 units, Worthington Biochemical LK003176) for 30 min at 37°C. Cells were maintained in neurobasal medium (Gibco 21103-049) containing 2% B27 supplement (Gibco 17504-044), 1% Glutamax (Gibco 35050-038), and 1% penicillin/streptomycin (Sigma P4333). Neurons were transduced with recombinant AAV at DIV3 and maintained for 12-14 days. Viral transduction was performed in triplicates and viral knockdown assessed in ≥ 2 independent experiment.

Biochemistry

Cortical neurons were lysed in lysis buffer containing 20mM Tris pH8.0, 100mM NaCl, 1mM EDTA, 1% Triton X-100, 0,2% SDS, 2mM DTT, and complete protease and phosphatase inhibitors (Roche Applied Science). Affinity-purified anti-NL3 and NL2 isoform-specific antibodies were previously described[3]. The following commercial available antibodies were used: mouse anti-tubulin (DHSB, Ab ID: AB_2315513 1:10000), mouse anti-synaptotagmin (Synaptic Systems, Cat. Nr. 105 011, 1:2000),

rabbit anti-NeuN (Abcam, ab177487, 1:2000). Immunoblotting was done with HRP-conjugated secondary antibodies and Pierce ECL Western Blotting Substrate. Signals were acquired using an image analyzer (Bio-Rad, ChemiDoc MP Imaging System and Li-Cor, Odyssey) and images were analyzed using ImageJ.

VTA DA neuron stimulation during habituation/non-familiar exploration task.

For the experiments using optogenetic tools, VTA::DA^{ChR2} and VTA::DA^{eYFP} mice were implanted with an optic fiber above the VTA. All the mice underwent optogenetic bursting stimulation (5 pulses of 4ms at 20Hz with 500 ms between the beginning of each burst) in a cage similar to home cage for 15 minutes. The optogenetic stimulation was non-contingent to the presence of a social stimulus. The mice used for electrophysiological recordings were sacrificed 24 hours after and the brain was sliced for *ex vivo* experiments, while the mice used for behavior started the habituation phase 24 hours after the 1st stimulation session. The mice underwent this stimulation protocol every day for 4 days (from day 0 to day 3), 5 hours after the free social exposure. Laser power was controlled between each test to ensure an estimated 7 – 10 mW of power at the implanted fiber tip. To assess the fiber placement and the viral infection, experimental subjects were sacrificed at the end of the habituation phase and transcardially perfused as previously described.

Statistical Analysis of the 3-chamber task

According to the original developer of the 3-chamber task, this assay is a yes-or-no test in which animals display sociability/social novelty or they do not[4]. For the statistical analysis and the interpretation of the results we followed the same principles, with minor adaptations. According to Nadler et al., 2004, during the sociability phase of the task, experimental subjects maintain higher time of interaction with a social stimulus compared to object for at least the first 10 minutes[5]. However, during the preference for social novelty, the experimental subjects maintain higher interaction time with a novel mouse compared to the familiar one only during the first 5 minutes of the test, while this difference is lost at later time points. For this reason, we decided to bin the time-course of both social preference and social novelty phases in two-time intervals: first 5 minutes (0-5) and last five minutes (5-10). For the time in chamber analysis, we performed a RM one-way ANOVA on the time spent in social, center or object chamber

or on time spent in novel, center of familiar stimulus chamber for the two-time intervals, separately. If the ANOVA analysis gave a $P < 0.05$, we proceeded with multiple comparisons between of social *vs* center and social *vs* object (for the two-time bins, separately) or non-familiar *vs* center and non-familiar *vs* familiar (for the two- time bins, separately) by applying the Holm-Sidak correction. For the time sniffing, we performed a RM two-way ANOVA followed by Bonferroni post-hoc test. By performing this within-group analysis, we considered the animals to express sociability or preference for social novelty if they spent more time in the social/non-familiar social chamber compared to the object/familiar mouse chamber and if they were engaged for longer time in social interaction or non-familiar social interaction compared to object and familiar stimulus interaction, respectively. Additionally, we calculated social novelty index, or preference score S2-S1 or S4-S3, to allow between group comparisons[6] after a significant RM two-way ANOVA ($P< 0.05$ for main effects and interaction) followed by Bonferroni post-hoc test.

References

1. Wess, J., Nakajima, K. & Jain, S. Novel designer receptors to probe GPCR signaling and physiology. *Trends Pharmacol. Sci.* **34,** 385–392 (2013).
2. Yang, M. & Crawley, J. N. Simple behavioral assessment of mouse olfaction. *Curr Protoc Neurosci* **Chapter 8,** Unit 8.24–8.24.12 (2009).
3. Budreck, E. C. & Scheiffele, P. Neuroligin-3 is a neuronal adhesion protein at GABAergic and glutamatergic synapses. *Eur. J. Neurosci.* **26,** 1738–1748 (2007).
4. Silverman, J. L. *et al.* GABAB Receptor Agonist R-Baclofen Reverses Social Deficits and Reduces Repetitive Behavior in Two Mouse Models of Autism. *Neuropsychopharmacology* **40,** 2228–2239 (2015).
5. Nadler, J. J. *et al.* Automated apparatus for quantitation of social approach behaviors in mice. *Genes Brain Behav.* **3,** 303–314 (2004).
6. Won, H. *et al.* Autistic-like social behaviour in Shank2-mutant mice improved by restoring NMDA receptor function. *Nature* **486,** 261–265 (2012).

Supplementary Statistical table

**Fig.1c**	Friedman test followed by Dunn's test for planned multiple comparisons
**Fig.1d**	Wilcoxon test
**Fig.1e**	RM two-way ANOVA followed by Bonferroni post-hoc test
**Fig.1f**	Kruskal-Wallis test followed by Dunn's multiple comparisons test
**Fig.2b**	RM one-way ANOVA followed by Holm-Sidak post-hoc test for planned comparisons
**Fig.2c**	RM one-way ANOVA followed by Holm-Sidak post-hoc test for planned comparisons
**Fig.2d**	RM one-way ANOVA followed by Holm-Sidak post-hoc test for planned comparisons
**Fig.2e**	RM two-way ANOVA followed by Bonferroni post-hoc test
**Fig.2f**	RM two-way ANOVA followed by Bonferroni post-hoc test
**Fig.2g**	RM two-way ANOVA followed by Bonferroni post-hoc test
**Fig.2h**	One-way ANOVA followed by Bonferroni post-hoc test for planned comparisons
**Fig.2i**	RM two-way ANOVA followed by Bonferroni post-hoc test for planned comparisons
**Fig.3c**	Paired t-test
**Fig.3d**	Paired t-test
**Fig.3e**	Paired t-test
**Fig.3f**	Friedman test
**Fig.3g**	Friedman test
**Fig.3h**	Friedman test
**Fig.3i**	Kruskal-Wallis test followed by Dunn's test for planned comparisons
**Fig.3k**	RM two-way ANOVA followed by Bonferroni post-hoc test for planned comparisons
**Fig.4b**	RM two-way ANOVA followed by Bonferroni post-hoc test
**Fig.4c**	Unpaired t-test
**Fig.4d**	RM two-way ANOVA followed by Bonferroni post-hoc test
**Fig.4e**	Mann-Whitney test
**Fig.4g**	Paired t-test
**Fig.4h**	Unpaired t-test
**Fig.4i**	Unpaired t-test
**Fig.4j**	Unpaired t-test
**Fig.4l**	RM two-way ANOVA followed by Bonferroni post-hoc test for planned comparisons
**Fig.5d**	RM two-way ANOVA followed by Bonferroni post-hoc test for planned comparisons
**Fig.5f**	RM two-way ANOVA followed by Bonferroni post-hoc test
**Fig.5g**	Unpaired t-test
**Fig.5i**	Paired t-test
**Fig.5j**	Unpaired t-test
**Fig.5k**	Mann-Whitney test
**Fig.5l**	Mann-Whitney test
**Fig.6a**	One-way ANOVA followed by Bonferroni post-hoc test for planned comparisons
**Fig.6b**	One-way ANOVA
**Fig.6c**	One-way ANOVA followed by Bonferroni post-hoc test for planned comparisons
**Fig.6d**	One-way ANOVA followed by Bonferroni post-hoc test for planned comparisons
**Fig.7c**	RM two-way ANOVA
**Fig.7d**	RM two-way ANOVA
**Fig.7g**	Mann-Whitney test
**Fig.7h**	RM two-way ANOVA
**Fig.8a**	One-way ANOVA followed by Bonferroni post-hoc test
**Fig.8b**	Unpaired t-test
**Fig.S1b**	RM two-way ANOVA
**Fig.S1c**	One-way ANOVA followed by Bonferroni post-hoc test for planned comparisons
**Fig.S1d**	Paired t-test
**Fig.S1e**	RM two-way ANOVA
**Fig.S1f**	One-way ANOVA
**Fig.S2a**	RM one-way ANOVA followed by Holm-Sidak post-hoc test for planned comparisons
**Fig.S2b**	RM one-way ANOVA followed by Holm-Sidak post-hoc test for planned comparisons
**Fig.S2c**	RM one-way ANOVA followed by Holm-Sidak post-hoc test for planned comparisons
**Fig.S2d**	RM two-way ANOVA followed by Bonferroni post-hoc test

**Fig.S2e**	RM two-way ANOVA followed by Bonferroni post-hoc test
**Fig.S2f**	RM two-way ANOVA followed by Bonferroni post-hoc test
**Fig.S2g**	One-way ANOVA followed by Bonferroni post-hoc test for planned comparisons
**Fig.S3a**	RM two-way ANOVA by both factor
**Fig.S3b**	RM two-way ANOVA by both factor followed by Bonferroni post-hoc test
**Fig.S3c**	RM two-way ANOVA by both factor
**Fig.S3d**	RM two-way ANOVA by both factor followed by Bonferroni post-hoc test
**Fig.S3e**	RM two-way ANOVA by both factor
**Fig.S4a**	Friedman test followed by Dunn's post-hoc test for planned multiple comparisons
**Fig.S4b**	RM one-way ANOVA followed by Bonferroni post-hoc test for planned multiple comparisons
**Fig.S4c**	Wilcoxon test
**Fig.S4d**	Paired t-test
**Fig.S4e**	RM two-way ANOVA for within and between genotype followed by Bonferroni post-hoc test
**Fig.S4f**	RM two-way ANOVA by both factor followed by Bonferroni post-hoc test
**Fig.S4g**	RM two-way ANOVA by both factor followed by Bonferroni post-hoc test
**Fig.S5d**	RM two-way ANOVA followed by Bonferroni post-hoc test
**Fig.S5e**	Unpaired t-test
**Fig.S5f**	RM two-way ANOVA by both factor followed by Bonferroni post-hoc test
**Fig.S5g**	RM two-way ANOVA by both factor followed by Bonferroni post-hoc test
**Fig.S5h**	Friedman test followed by Dunn's post-hoc test for planned multiple comparisons
**Fig.S5i**	RM one-way ANOVA followed by Bonferroni post-hoc test for planned multiple comparisons
**Fig.S5j**	Paired t-test
**Fig.S5k**	Paired t-test
**Fig.S5l**	RM two-way ANOVA for within and between virus injection followed by Bonferroni post-hoc test
**Fig.S6c**	RM two-way ANOVA followed by Bonferroni post-hoc test
**Fig.S6d**	Mann-Whitney test
**Fig.S6e**	Friedman test followed by Dunn's post-hoc test for planned multiple comparisons
**Fig.S6f**	RM one-way ANOVA followed by Bonferroni post-hoc test for planned multiple comparisons
**Fig.S6g**	Wilcoxon test
**Fig.S6h**	Paired t-test
**Fig.S7a**	Unpaired t-test
**Fig.S7b**	One-way ANOVA
**Fig.S7d**	Wilcoxon test
**Fig.S7e**	Paired t-test
